# Chemical and biological characterization of vaccine adjuvant QS-21 produced via plant cell culture

**DOI:** 10.1016/j.isci.2024.109006

**Published:** 2024-01-26

**Authors:** Xiangmin Lv, Jesse Martin, Heather Hoover, Bishnu Joshi, Margaret Wilkens, David A. Ullisch, Thomas Leibold, John S. Juchum, Sanket Revadkar, Barbara Kalinovska, Justin Keith, Adam Truby, Gui Liu, Elaine Sun, John Haserick, Jon DeGnore, Joseph Conolly, Adrian V.S. Hill, John Baldoni, Charlotte Kensil, Dan Levey, Alexandra J. Spencer, Gilbert Gorr, Mark Findeis, Antoine Tanne

**Affiliations:** 1 SaponiQx, Lexington MA, USA; 2 Agenus, Inc, Lexington MA, USA; 3 Phyton Biotech GmbH, Ahrensburg, Germany; 4 Phyton Biotech LLC, 1503 Cliveden Avenue, Delta, BC V3M 6P7, Canada; 5 The Jenner Institute, Nuffield Department of Medicine, University of Oxford, Oxford, UK; 6 Hunter Medical Research Institute, School of Biomedical Sciences and Pharmacy, College of Health, Medicine & Wellbeing; Immune Health Program, New Lambton Heights, NSW, Australia

**Keywords:** natural product synthesis, medical biochemistry, cell, Phytochemistry, Bioactive plant product

## Abstract

Many vaccines, including those using recombinant antigen subunits, rely on adjuvant(s) to enhance the efficacy of the host immune responses. Among the few adjuvants clinically approved, QS-21, a saponin-based immunomodulatory molecule isolated from the tree bark of *Quillaja saponaria* (QS) is used in complex formulations in approved effective vaccines. High demand of the QS raw material as well as manufacturing scalability limitation has been barriers here. We report for the first-time successful plant cell culture production of QS-21 having structural, chemical, and biologic, properties similar to the bark extracted product. These data ensure QS-21 and related saponins are broadly available and accessible to drug developers.

## Highlights

• Transformative method for manufacturing QS-21 vaccine adjuvant
• Enhancing the sustainability of QS-21 vaccine adjuvant production
• Bioequivalence of plant cell culture and conventional bark extract QS-21 vaccine adjuvants
• Revolutionary *in vitro* platform to develop plant saponin-derived vaccine adjuvants

## Graphical abstract

**Figure:**
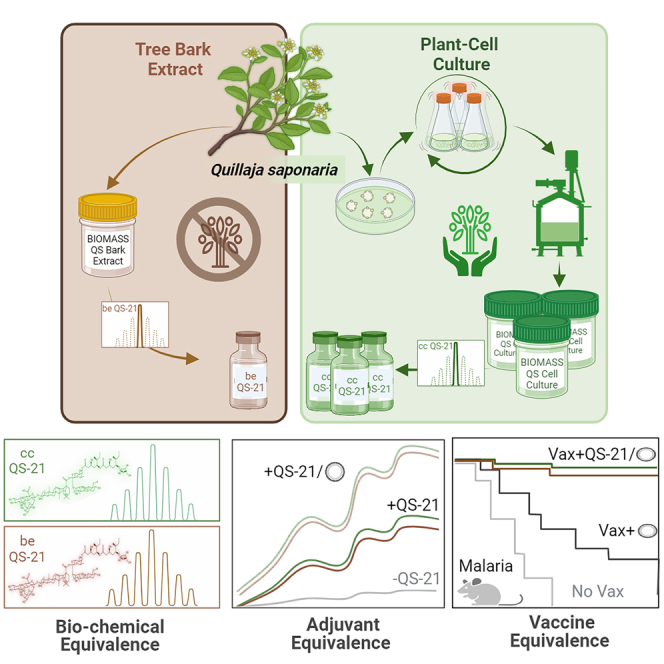

## Introduction

During the severe acute respiratory syndrome coronavirus 2 (SARS-CoV-2) pandemic, many structural and supply chain challenges related to vaccine design, development, manufacturing, and administration have been revealed.^1^ Adjuvants enhance immunogenicity by initiating and modulating both innate inflammation and adaptive immune responses. These components play a key role in determining the dynamic, breadth and depth of the acute vaccine response, as well as the quality and longevity of the memory immune response. Adjuvants are instrumental to precision vaccinology tailored to specific immune mechanisms, they drastically enhance antigen protective potency, and they significantly expand the therapeutic reach of vaccines including to neonates, elderly, and immune compromised individuals.^2^

Despite the widely acknowledged advantages of adjuvants, only a few sufficiently potent ones with acceptable toxicity levels are regularly being used. These include aluminum salts, oil-in-water emulsions containing squalene, *in vitro* assembled virosomes, or toll-like receptor (TLR) agonists and saponins formulated in liposomes or immune stimulating complexes (ISCOM).^3^

Among the most potent adjuvants used in licensed vaccines are those formulated with QS-21 saponin, a natural product mixture derived from the bark of the South American *Quillaja Saponaria* (QS) tree. Adjuvant System 01 (AS01) and the The Army liposome formulation with QS-21 (ALFQ) are liposomal formulation adjuvant mixture comprised of 3-*O*-desacyl-4′-monophosphoryl lipid A (MPLA), a derivative of Lipid A from *Salmonella minnesota* lipopolysaccharides (LPS; endotoxin) and QS-21, a saponin purified from the extract of the bark of the QS *Molina* tree.^4^^,^^5^^,^^6^^,^^7^ AS01 is utilized in several recently approved vaccines, including the first Respiratory Syncytial Virus (RSV) vaccine Arexvy, the first adjuvanted sub-unit malaria vaccine Mosquirix, and the herpes zoster vaccine Shingrix. Mosquirix, the first adjuvanted recombinant anti-malaria subunit vaccine, received positive opinion from the European Medicines Agency (EMA) in 2015 and a recommendation from the World Health Organization (WHO) in 2021 for use in children in Plasmodium falciparum endemic regions.^8^^,^^9^ Shingrix, an AS01 adjuvanted subunit vaccine targeting the VZV glycoprotein E (gE) has shown unprecedented effectiveness in elderly populations.^7^ Matrix-M is a differing ISCOM formulation of QS saponins fractions A and C (containing QS-21).^10^^,^^11^ This ISCOM was successfully used to adjuvant the first COVID-19 subunit authorized nanoparticle vaccine,^12^ NVX-CoV2373,^13^^,^^14^^,^^15^^,^^16^^,^^17^ and a novel clinical stage subunit Malaria vaccine such as R21.^18^^,^^19^

QS saponins, including QS-21, were originally identified and isolated from the aqueous extract of the bark of QS in the late 1980s.^20^ Today, they are primarily isolated from bark sourced from trees grown in the foothills of the Chilean Andes Mountain range.^21^ These intricate natural products consist of a hydrophobic triterpene core, an aliphatic sidechain, and complex, defined carbohydrate functional groups. In the quest for alternative manufacturing methods, semi-synthetic chemistry, biochemistry^22^^,^^23^^,^^24^^,^^25^^,^^26^^,^^27^ and agricultural plant microculture or sapling microfarming^28^^,^^29^ have been explored. A key breakthrough was recently made by identifying the crucial enzymatic pathways involved in the synthesis of QS-21-related immune modulatory saponins.^30^^,^^31^ This discovery opens up promising opportunities to use alternative platforms that combine bioengineering and semi-synthetic chemistry to boost the production of more sustainable QS-21 related saponin, while also enabling the engineering of novel adjuvants. Despite all being promising none of these methods have yet been approved nor have demonstrated suitable sustainability and cost-effectiveness. An alternative approach is plant cell culture. QS cell suspension fermentation has the potential to provide scalable, reproducible QS-21 production as cell biomass, biomass extract and purified individual saponins.^32^^,^^33^

Here, we demonstrate plant cell culture of wild-type QS that maintains secondary metabolic pathways, with composition profiles similar to saponins extracted from Chilean-grown trees. QS-21 isolated from our suspension plant cell cultures (cell culture QS-21; ccQS-21) is compositionally, chemically similar to, and biologically equivalent to QS-21 isolated from tree bark extract (beQS-21) as determined by chemical analysis, *in vitro* inflammatory activity and preclinical vaccine adjuvanticity using ovalbumin, shingles, and malaria subunit vaccine models. In addition to QS-21, this new technology offers alternative, scalable manufacturing methods to produce other purified saponins and fractions of interest to vaccine developers.

## Results

### Establishing *QS* plant cell cultures and production of ccQS-21 equivalent to beQS-21

**Figure 1:**
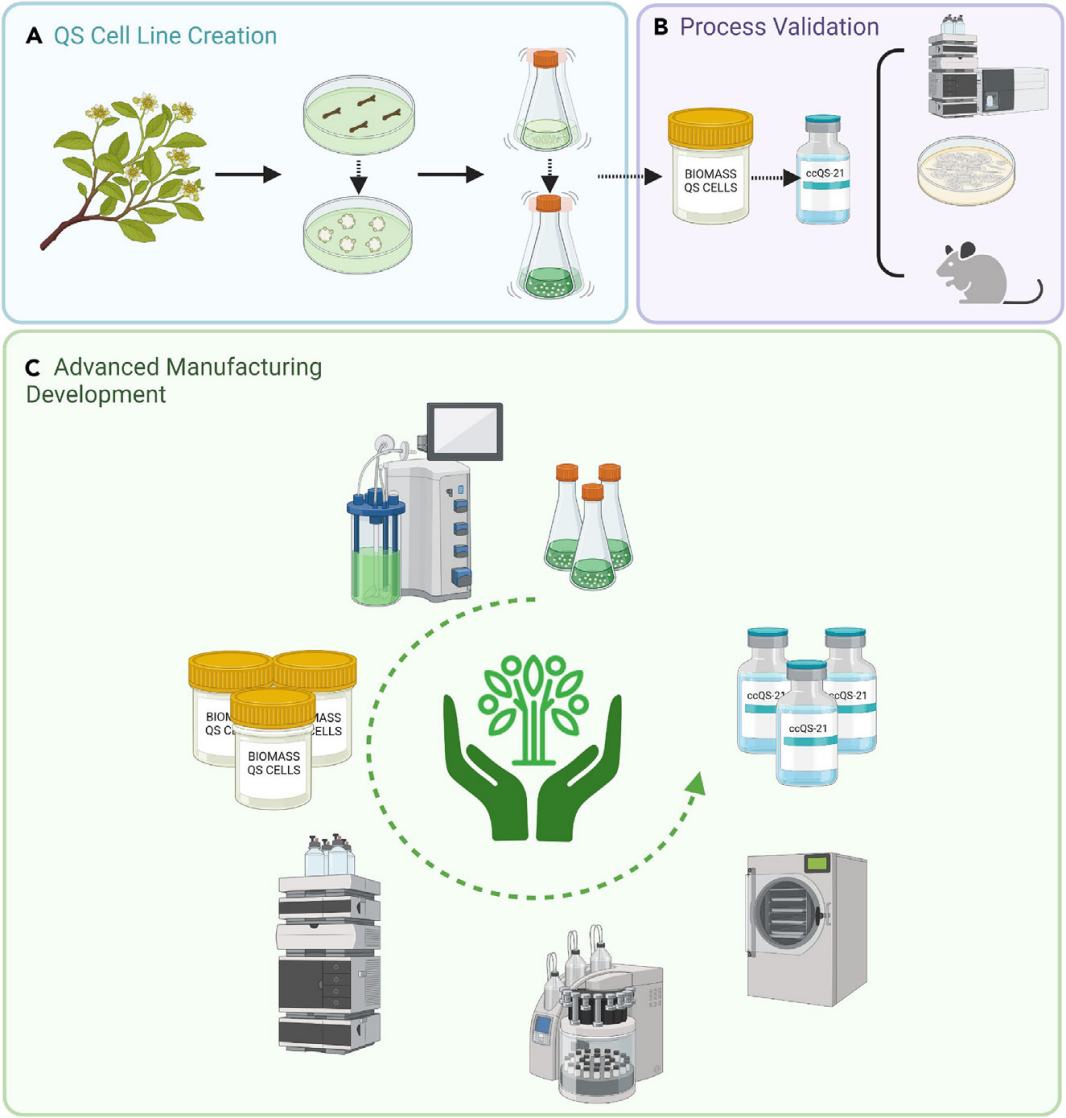
Summary scheme demonstrating key steps of a sustainable manufacturing process to generate high quantities of QS saponin derived adjuvant using QS plant cell culture (A) Plant cells were initially expanded on solid base from stems isolated from different *Quillaja saponaria* trees in Chile. Then, *in vitro* plant cell culture was developed from solid callus to liquid suspension. Expanding cell cultures have been selected based on their profile of production of the QS-21 saponin fraction and went through an upstream process development variation in induction protocols to maximize biomass and saponin production. (B) The QS-21 isolated from plant cell culture process has been characterized to demonstrate its analytical, biochemical and biological comparability with QS-21 isolated from bark extract. (C) Cell culture of QS-21 is now being translated into a scalable and sustainable Good Manufacturing Process. Selected and validated cell lines are now being used in a scalable plant cell fermentation process to produce biomass. To isolate ccQS-21, similar methods of downstream processing used to produce clinical grade GMP beQS-21 composing the QS-21 Stimulon formulation are being implemented to extract and purify high yields of ccQS-21.

**Figure 2:**
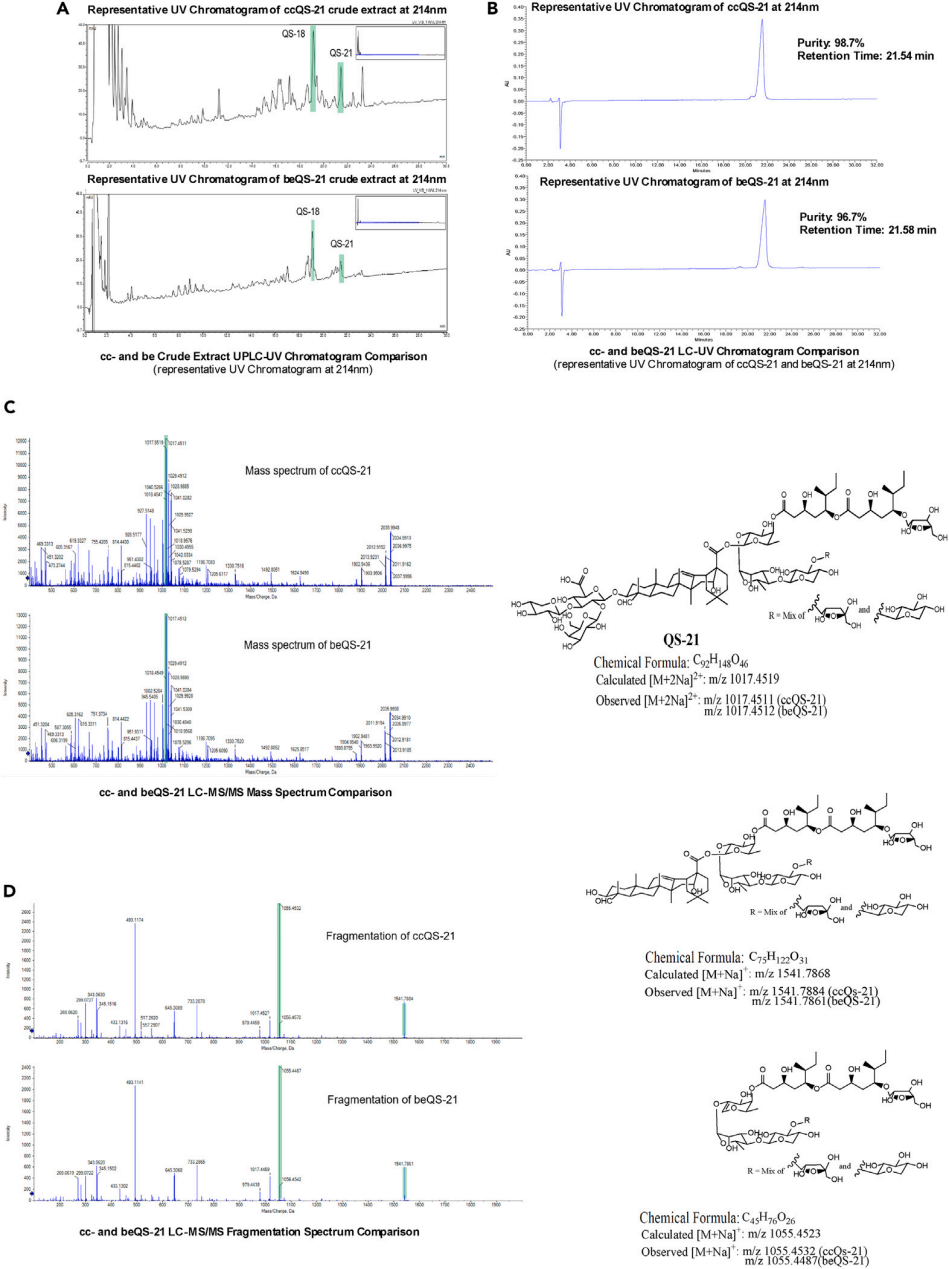
Analytical characterization of a representative development batch of ccQS-21 versus a reference batch of beQS-21 (A) UPLC-UV chromatogram detection demonstrating similar profile for cc and be crude extracts. (B) HPLC-UV chromatogram detection demonstrating similar retention time and purity of ccQS-21 and beQS-21. (C and D) The mass spectrometric (MS) fragmentation pattern of ccQS-21 is the same as beQS-21. The QS-21 1017.5 [M+2Na]^2+^ m/z parent ion (exact mass calculated for C_92_H_148_O_46_Na^+^_2_ as [M+2Na]^2+^: 1017.4519; observed: 1017.4512 [be], 1017.4511 [cc]) was subjected to MS/MS analysis and the same pattern of characteristic fragments was observed corresponding to the loss of the 3-*O*-linked trisaccharide producing a 1541.8 m/z ion (exact mass calculated. for C_75_H_122_O_31_Na^+^ as [M+Na]^+^: 1541.7868; observed: 1541.7861 [be], 1541.7884 [cc]), and the elimination of quillaic acid from the remaining glycoside to form an apparent alkoxy enol ether resulting in a 1055.5 m/z ion (exact mass calculated for C_92_H_148_O_46_Na^+^ as [M+Na]^+^: 1055.4523; observed 1055.4487 [be], 1055.4532 [cc]).

Authenticated wild-grown cuttings of mature, healthy QS trees were harvested in the Andean foothills in the vicinity of Santiago, Chile (Figures 1 and S1). Several stem explants were successfully grown on solid medium to generate callus cultures. Calli with visually superior growth properties were transferred to the growth medium to produce suspended mixed cell cultures. Small scale extraction of saponins from the mixed cell suspensions was performed and analyzed by high-performance liquid chromatography (HPLC) for the presence of saponins. Of the 23 mixed cell cultures created from several cuttings, 14 produced QS-21, typically in the fractions of mg/L range. The HPLC profile of the cell culture saponin fraction broadly resembled that observed in extracts of QS bark (I.E. Figure 2A). Several suspension cultures showed significant QS-21 production based on the saponin composition of their crude extract being comparable with bark extract (I.E. Ratio QS-X/QS-21). A single culture exhibiting both efficient growth and a higher QS-21 titer was selected for scale up. For the initial pilot proof of concept batch (Figures 1B and S1), three rounds of growth and induction were undertaken, 26 L of total culture volume produced, ∼2.6 kg of fresh weight of biomass which upon lyophilization afforded about 250 g of dry weight biomass containing an average concentration of QS-21 of 2,700 ppm. Following elution and a series of reverse-phase chromatographic steps, 24 mg of plant cell culture derived QS-21 was isolated, yielding a production level at this early stage of approximately 0.9 mg/L. Scalability and production yields subsequently improved across multiple development batches, which were analytically characterized with beQS-21 by HPLC coupled with ultraviolet (UV; Figures 2A and 2B) and mass spectrometric (MS; Figures 2C and 2D) detectors and shown to be equivalent by retention time and parent ion mass. Higher resolution HPLC of ccQS-21 confirm the presence of QS-21 V1, V2, minus pentose V1 and V1 as well as other congeners seen in beQS-21. Both proton (^1^H) (Figure S2A) and carbon-13 (^13^C) nuclear magnetic resonance (NMR) spectroscopy (Figure S2B) further confirmed the comparable structure of both QS-21. Multiple ccQS-21 batches were characterized using standard purity and identity. The integration of 4 analytical methods consistently indicates plant cell QS21 equivalence and demonstrate that the upstream cell culture process and downstream processing produce QS-21 that is similar to that isolated from bark extract, as described in the literature, and approved for clinical use.

### ccQS-21 and beQS-21 have conserved biochemical, biological, and immune modulatory properties

**Figure 3:**
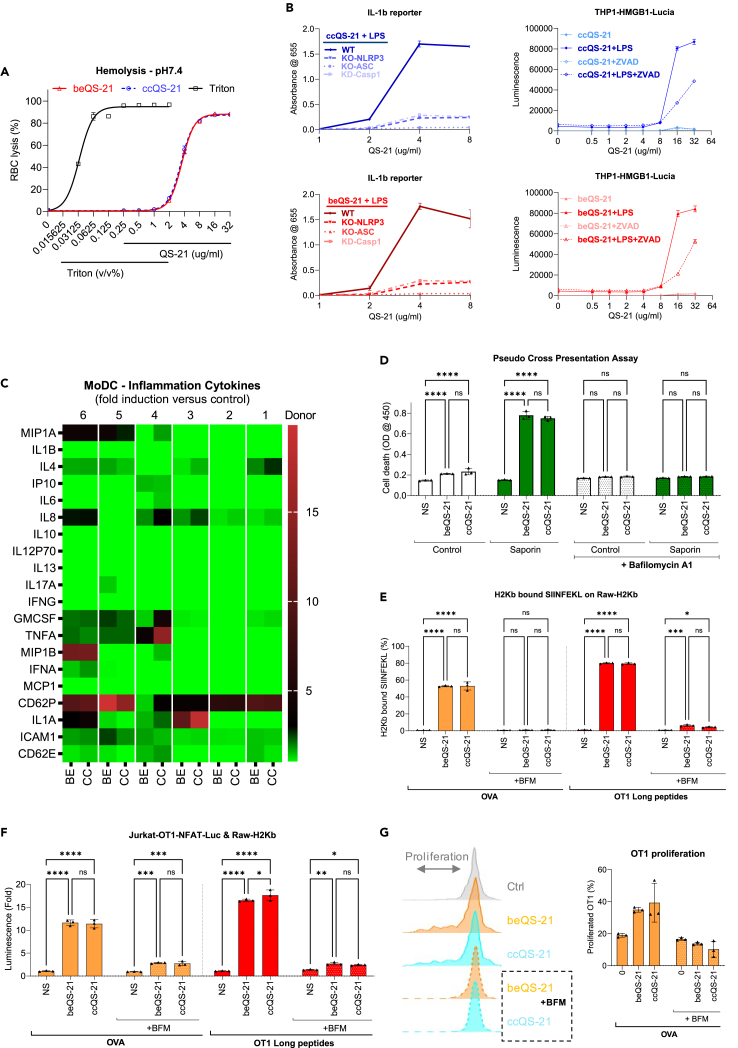
*In vitro* biological characterization; ccQS-21 and beQS-21 equivalently induce hemolysis, trigger the innate immune cell stress response, and enhance antigen processing and presentation to enhance the potency of the vaccinal immune synapse (A) Upon incubation with human red blood cells both beQS-21 and ccQS-21 retain equivalent saponin-specific hemolytic properties at pH-7.4 with matching half maximal effective concentration (EC50). (B) Upon pre-conditioning of THP1 human myeloid cells with a TLR4 agonist (LPS), both beQS-21 and ccQS-21 induce inflammasome-dependent secretion of IL-1β and the production of alarmins associated with pyroptosis such as HMGB1. Both IL-1β secretion and pyroptosis can be regulated by the canonical inflammasome pathway and can be suppressed by the disruption of the genes encoding for NLRP3, ASC and Caspase-1 or by the use of Caspase-1 inhibitor, ZVAD. (C) Both beQS-21 and ccQS-21 can comparably induce a broader inflammatory response *in vitro*. The breadth of the inflammatory response induced by the stimulation of human granulocyte macrophage colony stimulating factor (GMCSF) and IL-4 differentiated Monocyte derived Dendritic Cells (Mo-DCs) using both QS-21 was assessed with multiplex cytokine quantification. Both beQS-21 (BE) and ccQS-21 (CC) induce the same inflammatory signature which is further enhanced by formulating with TLR4 agonist. (D) Pseudo cross-presentation assay using cell viability to demonstrate that beQS-21 and ccQS-21 equivalently induce cytotoxicity through the regulation of the cytosolic translocation from the phagolysosome (e.g., as of the indicated toxin: saporin), a critical process for antigen cross presentation. (E) Flow cytometry analysis demonstrating that both QS-21 equivalently enhance large antigen processing and cross-presentation on MHC-I using a T cell receptor (TCR)-like antibody against the H-2K^b^-SIINFEKL complex. (F) *In vitro* antigen cross-presentation assay demonstrating that ccQS-21 and beQS-21 equivalently enhance antigen processing and minimal epitope cross-presentation to activate transgenic T cells with matched OT1 TCR to express NFAT luciferase reporter. (G) OT1 T cell proliferation shows ccQS-21 and beQS-21 equivalently enhance antigen cross-presentation to induce the proliferation of primary T cells with matched OT1 TCR. OVA: Ovalbumin, OT1 long peptide: 29mer peptide including SIINFEKL, BFM: Bafilomycin A1, inhibiting the vacuolar type H^+^-ATPase (v-ATPase) to transfer protons into the lysosome.

Both exhibit similar membrane disruption, as evaluated by their titration in hemolysis assays (Figure 3A). Inflammasomes are sets of receptors and sensors that regulate cellular stress response through the induction of caspase-dependent danger associated molecular patterns (DAMPs) and inflammatory cytokine release, in addition to the activation of pyroptosis, an immunogenic form of cell death.^34^ QS-21 activates the canonical Nucleotide-binding and Leucine-rich Repeat Protein-3 (NLRP3) inflammasome leading to pro-caspase-1 activation and the secretion of inflammatory pro-cytokines such as interleukin 1 beta (IL-1β) and interleukin 18 (IL-18).^35^ This activity was observed equally in ccQS-21 and beQS-21 demonstrating the equivalent activation of caspase-1 and LPS dependent production of IL-1β and IL-18. Both QS-21 sources triggered the cellular innate immune response through the canonical NLRP3, Apoptosis-Associated Speck-Like Protein Containing CARD (ASC), caspase-1 inflammasome (Figure 3B). We also assessed dependence on NLR Family CARD Domain Containing 4 (NLRC4) and the non-canonical caspase-4-dependent inflammasome, showing that the QS-21 mechanism of action is conserved between ccQS-21 and beQS-21 and mostly restricted to the canonical NLRP3 inflammasome. In LPS-preconditioned myeloid cells, both sources of non-formulated QS-21 induce alarmin High-Mobility Group Box 1 (HMGB1) release in a comparable dose-dependent and inflammasome-dependent manner, which can be efficiently suppressed by caspase-1 inhibitors (Figure 3B). We demonstrated that both ccQS-21 and beQS-21 equivalently induced the secretion of a broad spectrum of innate inflammatory cytokines and chemokines, both in the absence and presence of TLR4 conditioning to prime the inflammasome pathway (Figures 3C and S3B). The cytokine profile observed was similar to previous studies.^35^

QS-21’s membrane disruption properties enable it to contribute to antigen cross-presentation through the enhancement of passive uptake through the cellular membrane and by enhancing the leakage of antigens from the phagolysosome to the cytoplasmic compartment.^36^^,^^37^ Next, we demonstrated that both ccQS-21 and beQS-21 have similar membrane disruption properties (Figure 3A) which translate into an equivalent ability to regulate the translocation of macromolecules from the endo-phagolysosome to the cytoplasm, leading to the enhancement of antigen processing and cross-presentation on major histocompatibility complex I (MHC-I) to trigger CD8 T cell responses (Figures 3D–3F).

When formulated in liposomes, both sources of QS-21 remained comparable. The liposome formulation modulates some biological properties of QS-21, significantly reducing hemolytic properties (Figure S3A) and modulating pyrogenic properties in specific cell lines. However, when using primary human myeloid cells, both formulated QS-21s retained their ability to induce inflammatory responses (Figure S3B) and efficiently upregulate the antigen-presenting cell (APC) functions, such as antigen cross-presentation (Figures S3C‒S3F).

### ccQS-21 and beQS-21 have conserved adjuvant immune priming and conditioning properties *in vivo*

**Figure 4:**
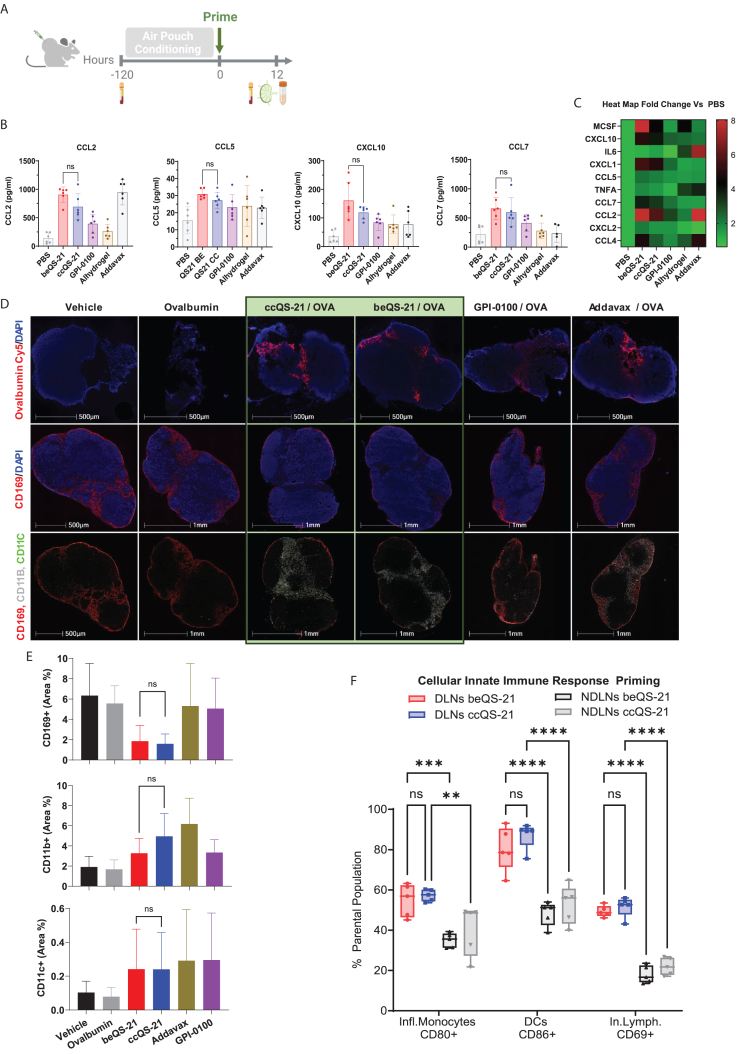
*In vivo* biological characterization; both beQS-21 and ccQS-21 can comparably induce an inflammatory response *in vivo* and enhance the draining lymph nodes (DLN) priming (A) Mice were conditioned using the air pouch experimental model to characterize the site of injection and DLNs innate immune response 12 h post-stimulation. (B and C) Both QS-21 induces the same spectrum of inflammatory cytokine response at the site of injection (B-C) and systemically (Figure S4A). (D and E) Immune phenotyping by multiplex fluorescent Immunohistochemistry (IHC) of DLNs after overnight (O/N) exposure to adjuvants revealed that both beQS-21 and ccQS-21 comparably enhance the trafficking of antigen (OVA) to the DLNs and induce specific DLN reorganization by targeting CD169+ subcapsular macrophages and inducing the infiltration of myeloid cells, and the activation of B cells in the germinal centers (Figure S4C). (F) Immune phenotyping by flow cytometry of DLNs and non-draining lymph nodes (NDLNs) after O/N exposure to adjuvants confirmed that both beQS-21 and ccQS-21 can equivalently induce innate immune myeloid and lymphoid cell recruitment and activation in the vaccinal DLNs. OVA, Ovalbumin.

The equivalence of ccQS-21 and beQS-21 adjuvant properties was further examined *in vivo*. In mammals, subcutaneously injected QS-21 has a localized effect and predominantly induces an innate immune priming response at the site of injection and in the vaccinal draining lymph nodes (vDLNs). Using an Air pouch model, ccQS-21 and beQS-21 induced similar innate immune inflammatory cellular and cytokine-chemokine responses at the site of injection, systemically, and in the vDLNs (Figures 4 and S4). Both types of QS-21 induce a panel of cytokines and chemokines (Figures 4B and 4C) mostly associated with the recruitment, trafficking, and activation of leukocytes; more specifically innate myeloid cells.^38^^,^^39^ This cytokine profile can be further biased toward T_H_1 response priming upon combination with a TLR4 agonist. Upon injection, QS-21 quickly distributes to the DLNs. When formulated in ISCOMs or liposomes^40^^,^^41^ it accumulates in subcapsular macrophages and progressively induces a restructuring of the DLNs, including the depletion of CD169+ macrophages and massive accumulation of myeloid and innate lymphoid cells. We demonstrated that both unformulated ccQS-21 and beQS-21 can equivalently target DLN subcapsular macrophages inducing a unique and specific reorganization of those CD169+ cells as compared to other adjuvants including the saponin-derived GPI-0100, aluminum hydroxide gel, and AddaVax (Figures 4D and 4E). Both ccQS-21 and beQS-21 induced a similar accumulation of antigens, recruitment, and activation of monocytes, neutrophils, and innate lymphoid cells (Figures 4D–4F and S4B), in addition to triggering an acute priming of the local B cell response in the germinal centers (Figure S4C).

### ccQS-21 and beQS-21 have equivalent adjuvant potencies and increase antigen immunogenicity

**Figure 5:**
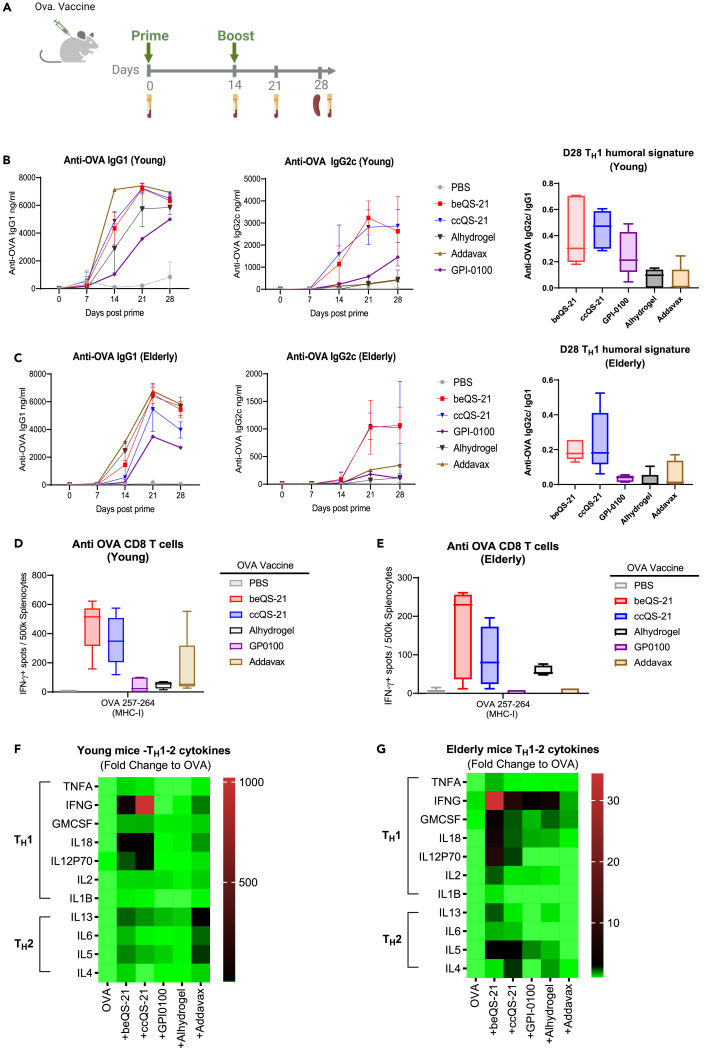
*In vivo* biological characterization; ccQS-21 and beQS-21 have equivalent vaccine adjuvant potency in young and elderly mice (A) Prime-boost (day 0/day 14) approach used in young versus elderly C57BL6/J mice with sub-unit ovalbumin vaccine model. (B and C) Both ccQS-21 and beQS-21 can equivalently induce a broad anti-ovalbumin T_H_1 humoral response with higher ratios of anti-OVA Ig2c versus IgG1 as compared to reference adjuvants in young (B) and elderly (C) mice. (D‒G) the *ex vivo* anti-ovalbumin T cell response demonstrated that both ccQS-21 and beQS-21 enhance anti-ovalbumin immune response priming. (F and G) The *ex vivo* T cell restimulation cytokine signature further demonstrated an equivalent ability to trigger a T_H_1 biased response. These properties can further be enhanced by combining the beQS-21 and ccQS-21 with MPLA to potentiate the T_H_1 vaccinal response in young animals (Figure S5A).

The equivalence of *in vivo* priming adjuvant properties of ccQS-21 and beQS-21 were further examined through *in vivo* immunogenicity studies. Both QS-21 formulations enhanced the immunogenicity of a subunit experimental vaccine against ovalbumin when injected in both young and elderly animals (Figures 5 and S5). Both formulations triggered potent and unique T_H_1 inflammatory conditioning as compared to aluminum-hydroxide gel, AddaVax, and GPI-0100, which led to the activation of a potent CD8 T cell response (Figures 5D and 5E) and humoral response observed by an enhanced conversion from IgG1 to IgG2c production in C57BL/6 mice (Figures 5B and 5C). IgG2c was equivalent to IgG2a in BALB/c mice and to IgG1 in humans, all of which can trigger the activator fragment crystallizable receptor and mediate antibody-dependent cell toxicity and cell phagocytosis, respectively.^42^^,^^43^^,^^44^ Both QS-21 formulations prime a T_H_1/T_H_2 cytokine profile upon pan T cell *ex vivo* reactivation; the T_H_1 signature being extensively enhanced as compared to the reference adjuvants (Figures 5F and 5G). The overall T_H_1 profile was further enhanced by combining both QS-21 types with monophosphoryl lipid A (MPLA; TLR4 agonist; Figure S5). QS-21 uniquely retained its outstanding adjuvant properties in immune deficient, elderly animals (Figures 5C–5E and 5G) and it is anticipated that the efficacy in elderly animals would be even further enhanced by the pairing with MPLA, as evidenced by the efficacy of Shingrix in the elderly.^45^

### ccQS-21 and beQS-21 have equivalent adjuvant potency in mice further enhanced when formulated in an monophosphoryl lipid A-containing liposomal formulation

**Figure 6:**
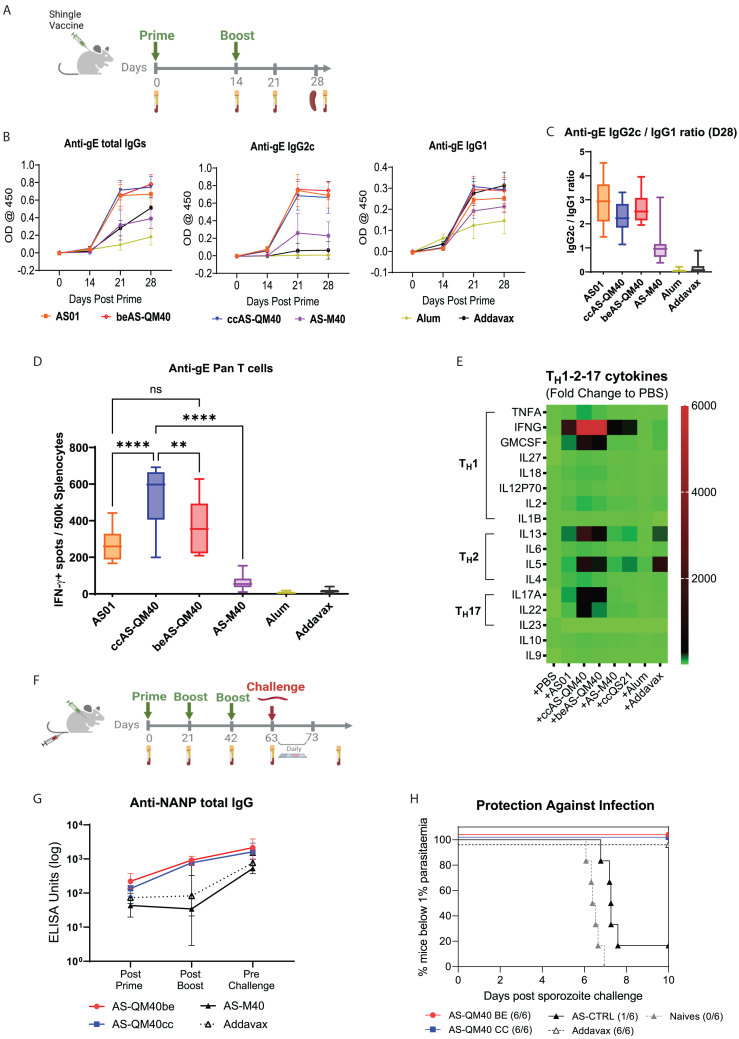
*In vivo* biological characterization; both ccQS-21 and beQS-21 formulated with MPLA in liposomes (AS01 mimetic formulations) efficiently adjuvant the R21 malaria vaccine and Shingrix vaccine (A) Using a prime-boost vaccination regimen with the Shingrix antigen (VZV gE) adjuvanted with the different test adjuvant formulations, we demonstrated that AS01 could be substituted by mimetic formulations using ccQS-21 and beQS-21. (B–E) All QS-21 formulations were equivalent at priming T_H_1 humoral (B-C) and a broad T_H_1-2-17 cellular (D-E) responses against the Shingrix antigen. (F) Using an experimental prime-boost-boost anti-malaria vaccination model with the R21 VLP vaccine followed by rechallenge with a surrogate malaria parasite, we further demonstrated the equivalent adjuvant potency of ccQS-21 and beQS-21. (G and H) Both ccQS-21 and beQS-21 prime an anti-CSP-NANP repeat humoral response leading to an efficient protection of 100% of the animal against parasite challenge, as measured by the daily parasitemia evaluation over a 10-day postexposure period. VLP, virus like particles; CSP, circumsporozoite protein; NANP, Immunogenic NANP repeats.

In the clinic, QS-21 potency and toxicity profiles have been improved through liposomal formulations containing MPLA and cholesterol.^28^^,^^35^^,^^46^^,^^47^ To further assess ccQS-21 and beQS-21 in a mouse model relevant to a human vaccine, both were formulated in a mimetic of AS01, the adjuvant system used in Shingrix, Mosquirix, and Arexvy, which has equivalent properties to the commercial AS01 adjuvant (Figures S3, 6A‒6E, S6A, and S6B). All had significantly reduced hemolytic properties at physiological pH (Figure S3A) and were less pyrogenic *in vitro*. All formulations retained immune modulatory properties by activating inflammasome-dependent and -independent inflammatory cytokine production (Figure S3B), and enhanced antigen cross-presentation (Figures S3C‒S3F) and T cell activation (Figure S3G). The main difference with unformulated QS-21 was related to the ability of the formulations to induce the inflammasome-regulated inflammatory cell death induction, pyroptosis, and release of DAMPs such as HMGB1. This is probably associated with the nonspecific cytotoxicity and membrane disruption properties of QS-21 being reduced upon liposome formulation. *In vivo*, synergy between MPLA and QS-21 in the liposomal AS01-like formulation increased the humoral and cellular T_H_1 response against an ovalbumin experimental subunit vaccine (Figures S5A‒S5C). We then tested the adjuvant efficacy of the liposomal formulation derived from both QS-21 types in the context of shingles and malaria vaccination. We demonstrated that the commercial AS01 formulation could be substituted in the Shingrix vaccine by either the beQS-21 or ccQS-21 derived AS01-like formulation to induce a specific T_H_1 humoral and T_H_1/T_H_2/T_H_17 cellular responses against the Glycoprotein E of Herpes Simplex Virus (gE HSV) zoster antigen (Figures 6B–6E). The ratio of IgG2c to IgG1 vaccine-induced immunoglobulin was superior to reference adjuvants (alum and squalene) and to the QS-21-free MPLA-liposome, demonstrating synergy between QS-21 and MPLA in the AS01-like formulation. Further, both types of QS-21 were benchmarked against a clinical-stage development adjuvanted recombinant subunit malaria vaccine R21 currently undergoing assessment in large Phase 3 trials. The AS01-like formulations containing QS-21 from either source enhanced the potency of an experimental malaria vaccine, R21, comprising the circumsporozoite protein (CSP) expressed in virus-like particles. R21 mixed with the liposomal QS-21 adjuvant formulations primed efficient humoral responses against CSP as compared to a reference squalene-derived adjuvant AddaVax and a reference liposome formulation containing MPLA alone (Figure 6G). Using the murine malaria challenge model, derived from *Plasmodium berghei* expressing CSP from the human parasite *Plasmodium falciparum*, we demonstrated that both formulations of QS-21 primed the same 100% protective immune response to that produced by the vaccine formulated with reference adjuvant (Figure 6H) and that were superior to control liposome-formulated MPLA alone. Finally, different ratios of QS-21 versus MPLA in the liposome demonstrated that both ccQS-21 and beQS-21 could be further reduced up to half the dose of saponin without affecting adjuvant potency, vaccine induced immunity, or efficacy (Figures S6A and S6B), demonstrating the flexibility of QS-21 and MPLA adjuvant formulation development and the potential for saponin dose-sparing.

## Discussion

QS saponin-including adjuvants QS-21, AS01, and Matrix-M are among the most potent and promising adjuvants available and have enabled significant medical breakthroughs such as the first approved or authorized recombinant vaccine against RSV, malaria, and COVID-19, and the most durable and efficient vaccine against Shingles to date.^2^^,^^3^ The limited supply of QS saponins is a barrier to their more widespread use. In this study, we describe the physicochemical and biological characterization of ccQS-21, derived from plant cell culture, as compared to beQS-21, from tree bark and demonstrate equivalent “adjuvant effects.” In a supply-constrained environment, ccQS-21 provides a path to increase the exploration of QS-21-enabled vaccines, while reducing costs and protecting the environment.

The mechanism of action of QS saponin adjuvants, such as QS-21, remains poorly understood. It has been proposed to be depot-independent, as opposed to alum. QS-21 immune modulatory properties are also independent of innate immune TLRs but may involve recognition by C-type lectins expressed by innate immune cells, and CD2 on T cells.^48^^,^^49^ At a cellular level, the QS-21 mechanism of action has been partially elucidated though the characterization of its synergistic effect with other adjuvants such as the TLR4 agonist phospholipid MPLA and the TLR9 agonist Cytosine-Phosphate-Guanine (CpG) dinucleotides. It has been demonstrated that QS-21 can stimulate APCs by inducing the formation of TLR-primed multi-protein complexes, namely inflammasomes.^35^ QS-21 also acts as an immunogenicity potentiator at the cellular level by enhancing antigen delivery and processing to increase cross-presentation on MHC-I.^5^^,^^50^^,^^51^

Our data demonstrate that ccQS-21 retains the critical innate immune priming properties of beQS-21, validating cell culture as a process to produce commercial grade ccQS-21. Among the key functions of immune inducers and antigen delivery potentiator adjuvants have the ability to enhance antigen processing and increase antigen immunogenicity through the enhancement of APC functions. We demonstrate that ccQS-21 retains APC-enhancing properties by inducing antigen leakage through the endo-lysosomal envelope, which favors antigen cross-presentation and in turn, increases the immunogenicity of macromolecular antigens. In addition, it primes efficient cellular innate immune response leading to the recruitment of key vaccine induced effector cells, as well as primes the adaptive immune response in DLNs. Both QS-21 formulations trigger the diversification of the humoral response beyond the generation of antibodies with neutralizing opportunities. The efficient activation of CD8 and T_H_1/T_H_2 cytokines are critical correlates to vaccine efficacy, more specifically against intracellular pathogens, cellular pathologies, and chronic latent diseases.

We demonstrate that both beQS-21 and ccQS-21 are compatible with AS01 and ALFQ-like formulations and induce efficient vaccine-induced responses against pathogens such as shingles and malaria. In murine studies, ccQS-21 formulations have demonstrated equivalence to commercial adjuvants and have shown promise as adjuvant dose sparing formulations. In aggregate, these data demonstrate that cell culture is a sustainable alternative to natural resources to produce QS-21 as an adjuvant. In this proof-of-concept approach, we show that the chemical, biochemical, and biophysical equivalence of bark extract and cell culture-derived QS-21 translate into conserved biological and adjuvant properties both *in vitro* and *in vivo*. Further, the use of cell culture to synthesize QS saponins opens the opportunity to systematically evaluate thousands of QS-21 congeners for adjuvant and other biological activities, thereby enabling further exploration of how this class of natural products and related compounds may impact medicine. With a better understanding of the genes and enzymes involved in the biosynthesis of these immune modulatory saponins,^30^^,^^31^ *in vitro* cultures such as our cell culture method will help unlock the discovery of key pathways to design novel adjuvants with advantageous tailored features. Looking forward, the use of engineered organisms or a complete chassis shift is expected to further facilitate the sustainable and scalable production of saponin-derived adjuvants and will enable precision vaccine development.

### Limitations of the study

The study presents a novel advancement in the sustainable production of the vaccine adjuvant QS-21 using plant cell culture, a method that may overcome the supply and scalability challenges associated with traditional extraction from Quillaja saponaria tree bark. The findings suggest that cell-cultured QS-21 (ccQS-21) is biochemically and functionally equivalent to bark-extracted QS-21 (beQS-21). However, the study shares only limited comprehensive data on manufacturing optimization and the challenges of large-scale production, such as maintaining saponin profile purity and yields. Critical information on process variable’s impact on such things as chromatography purification and consistent composition of QS-21 structural variants and the potential development of cell line banks could not be extensively discussed. In fact, details on know-how and proprietary preparative purification methods had to remain undisclosed at this stage, which may limit the understanding of the scalability of the production process. The study is yet discussing results based the characterization of a limited number of development and early stage GMP batches. It does only compare to standard QS-21 manufacturing methods and does not present a comparative analysis versus over other plant tissue or alternative organism culture technologies for QS-21 production. Finally, because of the lack of depth of information shared in this first report, it only provides a limited insight on the economic and ecological benefits compared to bark extraction and other potential alternative methods.

## STAR★Methods

### Key resources table

**Table:** 

REAGENT or RESOURCE	SOURCE	IDENTIFIER
**Antibodies**		

PE anti-mouse H-2Kb bound to SIINFEKL Antibody	BioLegend	141604; RRID: AB_10895905
BD Horizon™ BUV563 Rat Anti-Mouse CD8a	BD Biosciences	748535; RRID: AB_2872946
CD4 Monoclonal Antibody (GK1.5), NovaFluor™ Blue 610-70S, eBioscience™	Invitrogen	M001T02B06; RRID: AB_2896688
PE anti-mouse CD69 Antibody	BioLegend	104508; RRID: AB_313111
Brilliant Violet 421™ anti-mouse CD25 Antibody	BioLegend	102043; RRID: AB_2562611
BD OptiBuild™ BUV395 Rat Anti-Mouse CD3e	BD Biosciences	740268; RRID: AB_2687927
BD Horizon™ BUV805 Rat Anti-CD11b	BD Biosciences	568345; RRID: AB_2941960

**Biological samples**		

Human Red Blood Cells 10% Washed Pooled Cells	Rockland Immunochemicals	R407

**Chemicals, peptides, and recombinant proteins**		

Lipopolysaccharides from Escherichia coli O26:B6 (LPS)	Sigma-Aldrich	L8274
Bafilomycin A1	Selleck Chemicals	S1413
Saporin from Saponaria officinalis seeds	Sigma-Aldrich	S9896
Cytochrome *c* from equine heart	Sigma-Aldrich	C2867
Ovalbumin	InvivoGen	vac-pova-100
OT1 long peptide	Agenus	N/A
SIINFEKL peptide	GenScript	RP10611
E7 peptide	Agenus	N/A
beQS21	Agenus, Desert King	N/A
ccQS21	Agenus, SaponiQx	N/A
AS01	GSK	NDC 58160-829-03
AS01-like formulations	Avanti Lipid	Customized
Pan-Caspase inhibitor - Z-VAD-FMK	InvivoGen	tlrl-vad
OT-II peptide	GenScript	RP10610
MPLA Synthetic VacciGrade	InvivoGen	vac-mpls

**Critical commercial assays**		

CD14 MicroBeads, human	Miltenyi Biotec	130-050-201
CD8a+ T cell Isolation Kit, mouse	Miltenyi Biotec	130-104-075
Inflammation 20-Plex Human ProcartaPlex™ Panel	Invitrogen	EPX200-12185-901
Cytokine & Chemokine 26-Plex Mouse ProcartaPlex™ Panel 1	Invitrogen	EPX260-26088-901
Cytokine & Chemokine 36-Plex Mouse ProcartaPlex™ Panel 1A	Invitrogen	EPX360-26092-901
Th1/Th2 Cytokine 11-Plex Mouse ProcartaPlex™ Panel	Invitrogen	EPX110-20820-901
Th1/Th2/Th9/Th17/Th22/Treg Cytokine 17-Plex Mouse ProcartaPlex™ Panel	Invitrogen	EPX170-26087-901
Mouse anti-OVA IgG2c Antibody Assay Kit	Chondrex	3029
Mouse Anti-OVA IgG1 Antibody Assay Kit	Chondrex	3013
Mouse Anti-OVA IgG Antibody Assay Kit	Chondrex	3011
BD™ ELISPOT Mouse IFNg ELISPOT Set	BD Biosciences	551083
CytoTox 96® Non-Radioactive Cytotoxicity Assay	Promega	1780
Nano-Glo® Luciferase Assay	Promega	N1120

**Experimental models: Cell lines**		

THP1-Null2	InvivoGen	thp-nullz
THP1-KO-ASC	InvivoGen	thp-koascz
THP1-KO-NLRP3	InvivoGen	thp-konlrp3z
THP1-def CASP1	InvivoGen	thp-dcasp1
THP1-Null	InvivoGen	thp-null
THP1-HMGB1-Lucia	InvivoGen	thp-gb1lc
HEK-Blue-IL-1b	InvivoGen	hkb-il1bv2
Raw264.7-H2Kb (Kelton, W. et al.^52^)	Sai T. Reddy’s Lab	N/A
Raw264.7-H2Db	Sai T. Reddy’s Lab	N/A
OT1 TCR-Jurkat-NFAT-Luc	Agenus	N/A

**Experimental models: Organisms/strains**		

C57BL/6-Tg(TcraTcrb)1100Mjb/J (OT-1) mouse	Jackson Laboratory	003831
C57BL/6J mouse	Jackson Laboratory	000664
Aged C57BL/6J mouse	Jackson Laboratory	000664 | Aged Black 6
BALB/cOlaHsd (BALB/c) mouse	Envigo/Inotiv	162
Plasmodium berghei sporozoites (spz) expressing P.falciparum CSP (Triller, G. et al.^53^)	Adrian V. S. Hill’s Lab	N/A
Anopheles stephensi mosquitoes	Adrian V. S. Hill’s Lab	N/A

### Resource availability

#### Lead contact

Further information and requests for resources and reagents should be directed to and will be fulfilled by the lead contact, Antoine Tanne (antoine.tanne14@gmail.com).

#### Materials availability

The material characterized in this manuscript “cell culture QS-21” could be obtained from SaponiQx (https://saponiqx.com/contact-us/) under specific supply or transfer agreement.

#### Data and code availability

• Data reported in this paper will be shared by the lead contact upon request.
• This paper does not report original code.
• Any additional information required to reanalyze the data reported in this paper is available from the lead contact upon request.

### Experimental model and study participant details

#### Cell lines and primary cells

In this study, all cell lines were screened for contamination according to the established laboratory practices at Agenus, Inc. and SaponiQx, utilizing the IDEXX BioAnalytics STAT-Myco or IMPACT tests. The transgenic cell lines were verified for authenticity by their suppliers, InvivoGen and ATCC.

THP1-Null2, THP1-KO-ASC, THP1-KO-NLRP3, THP1-def CASP1, THP1-Null, THP1-HMGB1-Lucia, HEK-Blue- IL-1b cell lines were purchased from Invivogen and maintained following the vendor recommendation. Raw264.7-H2Kb and Raw264.7-H2Db were a gift from Professor Sai Reddy at the ETH Zurich and were initially described in Kelton et al.^52^ Raw264.7-H2Kb Cells were cultured in Immune Cell Media (RPMI1640 media supplemented with 10% heat inactivated FBS, 2 mM Glutamine, 1X Non-Essential Amino Acids, 1 mM Sodium pyruvate, 10 mM HEPES, 55uM β-mercaptoethanol, and 1X Penicillin/Streptomycin). Clonal Jurkat NFAT Luc OT1 cells were generated from authenticated TCRβ-deficient Jurkat cells from ATCC. TCRβ-deficient Jurkat cells were first stably transfected with an NFAT-Nano Luciferase reporter system, and then transduced with a lentiviral vector encoding for the murine OT1 TCR. Jurkat cell line were maintained in Immune Cell Media. Primary human PBMCs were isolated from whole blood of different donors. CD14^+^ monocytes were isolated using MACS magnetic positive CD14^+^ cell selection method as described in the manufacture’s instruction (Miltenyi Biotec, Germany). Monocyte derived dendritic cells (MoDC) were differentiated with human GMCSF and IL-4 in Immune Cell Media for 5 Days from monocytes isolated from PBMCs.^54^^,^^55^ Human red blood cells were purchased from (Rockland Immunochemicals, US). Murine primary OT1 T cells were isolated from spleens and lymph nodes of transgenic OT1 mice (Jackson Lab, US) using a MACS magnetic negative CD8^+^ T cell selection method as described in the manufacture’s instruction (Miltenyi Biotec, Germany).

#### Animals experimentation

##### General

All studies were performed using female mice. For the standard immunization trials, 8-10-week-old C57BL/6J mice (sourced from Jackson Laboratory, USA) were utilized, while research involving older subjects employed 70-week-old C57BL/6J mice, also from Jackson Laboratory, USA. The malaria research utilized 6-10-week-old BALB/cOlaHsd (BALB/c) mice obtained from Envigo. The selection of female mice for these experiments is a widely accepted method in the fields of immunology and vaccine research. This preference is based on the greater consistency observed in the female immune response and the behavioral benefits that facilitate the conduct of such studies.

For the malaria study conducted at the Jenner Institute (Oxford, UK), mice were used in accordance with the UK Animal (Scientific Procedures) Act under project license number P9804B4F1 granted by the UK Home Office. Animals were group housed in IVCs under SPF conditions, with constant temperature and humidity with lighting on a 13:11 light-dark cycle (7a.m. to 8p.m.). For induction of short-term anesthesia, animals were anesthetized using vaporized IsoFlo. All animals were humanely sacrificed at the end of each experiment by an approved Schedule 1 method (cervical dislocation). Other immunization studies were conducted by Agenus, Inc. and SaponiQx with the Neosome Life Sciences, LLC (Lexington MA, US) and all procedures were reviewed and approved by IACUC and conform to the guidelines established by NeoSome Life Sciences for the ethical care and treatment of laboratory animals in accordance with the NIH animal research guidelines. For induction of short-term anesthesia, animals were anesthetized using vaporized IsoFlo. All animals were humanely sacrificed at the end of each experiment by an approved Schedule 1 method.

##### Malaria immunization

For the malaria study, BALB/cOlaHsd (BALB/c) (Envigo) mice of at least 6-week of age, were immunized intramuscularly (im) in the *musculus tibialis* with a total of 50μL of vaccine following a prime, boost, boost regimen at days 0, 21, 42. For preparation of R21 vaccine and QS-21 liposome mixtures, 0.5 μg of R21 (1.4 μL of prep) was added directly to the liposomes (48.6 μL). To enable a final 1:1 ratio of Addavax to R21, 0.5 μg of R21 was diluted in sterile PBS and mixed with an equal volume of Addavax (InvivoGen).

##### Sporozoite preparation and “malaria” challenge

*Plasmodium berghei* sporozoites (spz) expressing *P*.*falciparum* CSP under the control of the *P*.*berghei* CSP promoter ^53^ were isolated from salivary glands of female *Anopheles stephensi* mosquitoes around 21 days after feeding on a *P*.*berghei* blood stage infected donor mouse. Salivary glands were homogenized, spz counted under phase contrast microscopy, and 1000 *P*.*berghei* spz intravenously injected into recipient mice 21 days post complete vaccination. Mice were monitored daily from day 5 onwards by taking a thin blood film and staining with 5% Giemsa (Sigma Aldrich) to screen for the presence of schizonts within the red blood cells. Parasitaemia was calculated as the percentage of infected red blood cells per microscope field (100x objective), with at least five fields counted per mouse per day. Using linear regression, the time to 1% parasitaemia was calculated based on the y-intercept and slope of the line. Mice were humanely sacrificed by an approved Schedule 1 method after three consecutive positive blood films or parasitaemia over 1%. Sterile protection was defined as the complete absence of parasite in the blood until at least 10 days post rechallenge.

##### Air pouch

8-week female C57BL6/J mice (Jackson Lab, US) were used for the air pouch study. To create the pouch, 5 mL of 0.2 μM filtered sterile air was injected subcutaneously under anesthesia, followed by another 3mL of 0.2 μM filtered sterile air after 3 days to maintain the pouch. To induce inflammation, 100 μL of treatment vaccine including 25 μg Cy5-labeled ovalbumin (in house product at Agenus/SaponiQx) and indicated adjuvant (beQS-21 or ccQS-21: 5 μg/mouse, GPI-0100: 25 μg/mouse, Addavax: 50μL/mouse, Alhydrogel: 50μL/mouse) was injected into the pouch using a 25-gauge needle. After 12 h; the air pouch exudate was collected under euthanasia after injecting 1.0 mL PBS into the air pouch and swirling around. A small incision was made in the top of air pouch to allow harvest of the lavage solution which was then centrifuged at 500 g for 10 min. Air pouch supernatant was aliquoted off for Luminex analysis. The remaining air pouch cell pellet were processed to single cell suspension for flow analysis. Draining lymph nodes and non-draining lymph nodes were harvested and processed for flow analysis and fluorescent immunohistochemistry staining and imaging.

##### Ovalbumin immunization

For the ovalbumin mouse study, 8-week (Young group) and 70-week-old (Elderly group) female C57BL6/J were ordered from Jackson Lab. After rest, each mouse was vaccinated subcutaneously in the back of neck at day 0 (Prime) and Day 14 (Boost) using a vaccine including 25 μg ovalbumin (Invivogen, France) and the indicated adjuvant (beQS-21 or ccQS-21: 5 μg/mouse, GPI-0100: 25 μg/mouse, Addavax: 50 μL/mouse, Alhydrogel: 50 μL/mouse, MPLA: 2 μg/mouse). beQS-21 or ccQS-21 were in-house products at Agenus/SaponiQx. GPI-0100 was from Hawaii Biotech (Hawaii, US). Addavax, Alhydrogel, and MPLA were from Invivogen. Plasma was collected at day 0, 14, 21, 28 for anti-ovalbumin IgG2c/IgG1/IgGs ELISA detection. Splenocytes were harvested at day 28 for Elispot and Luminex cytokines analysis after *ex vivo* restimulation. Draining lymph nodes and non-draining lymph nodes were also harvested and processed for downstream analysis.

##### Shingrix immunization

8-week-old female C57BL6/J mice (Jackson Lab, US) were vaccinated subcutaneously in the back of neck at day 0 (Prime) and day 14 (Boost) using a vaccine including 5 μg recombinant gE protein (GSK, UK) and indicated adjuvant (AS01b: 50 μL/mouse, AS-QM40be: 50 μL/mouse, AS-QM40cc: 50 μL/mouse, AS-M40: 50 μL/mouse, Addavax: 50 μL/mouse, Alhydrogel: 50 μL/mouse). AS01b and gE were from GSK Shingrix vaccine. AS-QM40be, AS-QM40cc, AS-M40 were in-house products at Agenus/SaponiQx as describe above. Addavax and Alhydrogel were from Invivogen. Plasma was collected at day 0, 14, 21, 28 for anti-gE IgG2c/IgG1/IgGs ELISA detection. Splenocytes were harvested at day 28 for Elispot and Luminex cytokines analysis.

### Method details

#### QS-21

Tree bark extract QS-21 was either manufactured at Agenus Inc or at Desert king; both having similar analytical profile and adjuvant properties. Harvest of authenticated clippings of native Chilean *Quillaja saponaria* (QS) trees in the vicinity of Santiago, Chile and subsequent plant cell culture, extraction and preparative HPLC to produce cell culture-derived QS-21 was performed at Phyton Biotech, Ahrensburg, Germany. ^1^H-NMR and ^13^C-NMR spectra were acquired at Eremid Research Services Inc., Kannapolis, NC. **Analytical UPLC method.** Acquity UPLC BEH C18 column, gradient: 30%–50% of 0.05% TFA acetonitrile in 0.05% TFA water in 30 min, UV detector: 214 nm, flow rate: 0.3 mL/min **Analytical HPLC method**. Reverse-phase HPLC data were acquired on a Waters Alliance 2695 HPLC chromatography system equipped with quaternary pump, a UV detector set to 214 nm, and a column compartment maintained at ambient temperature. Chromatography was performed on a Vydac 4.6 × 250 mm, 5 μm C4 column (Catalog # 214TP54) using a binary gradient (mobile phase A: 0.15% v/v trifluoroacetic acid in water; mobile phase B: 0.15% v/v trifluoroacetic acid in acetonitrile). Following equilibration to 70:30 (% mobile phase A: % mobile phase B), sample was injected via the auto-sampler and the column was eluted with a linear gradient as described in Table S1.

#### HPLC-mass spectrometry

LC-MS and LC-MS-MS were performed on a Sciex TripleTOF mass spectrometer connected to an Agilent 1260 UPLC chromatography system equipped with binary pumps, a diode array UV detector and a column compartment maintained at 25°C. Chromatography was performed by the same HPLC method described above. Following the UV detection (monitoring at 214 nm); flow is directed to the mass spectrometer. Mass spectrometry parameters were set as listed in Table S2.

#### Nuclear magnetic resonance (NMR) spectroscopy

Proton and carbon-13 nuclear magnetic resonance (1D ^1^H and 1D ^13^C NMR) spectra were recorded on 600 MHz and 125 MHz NMR spectrometers respectively by Eremid Research (Kannapolis, NC). Briefly, bark extract and cell culture derived QS-21 samples were dissolved at 6.1 mg/mL concentration in NMR compatible solvent (D_2_O/ACN-d_3_) containing internal standard and transferred to an NMR tube for analysis. Chemical shifts were referenced to DSS-d6 (internal standard) at 0 ppm for ^1^H and ^13^C. Annotations was made based on resonance assignments described in Jacobsen et al.^50^ 2D-NMR spectra using COSY, HMBC, HSQC, and NOESY are provided as supporting material (Figure S2C).

#### QS-21 liposome formulation

Commercial AS01 was used as a benchmark formulation, containing 50 μg MPLA and 50 μg QS-21 in 500 μL produced by GSK with beQS-21 (QS Molina, fraction 21) licensed by GSK from Antigenics LLC, a wholly owned subsidiary of Agenus Inc., a Delaware, USA corporation. Experimental AS01-like formulations were generated by Avanti Lipid (USA) using Agenus and SaponiQx beQS-21 and ccQS-21. The liposome formulation consisted of 65.5 mol % DOPC (cat# 850375), 33.3 mol % synthetic cholesterol (cat# 700100), and 1.2 mol % MPLA (cat# 699800). The liposomes were emulsified to an average diameter of ∼100 nm and were then loaded with varying concentrations of beQS-21 or ccQS-21. All formulation are described in Table S3.

#### HMGB1-lucia reporter assay

THP1-HMGB1-Lucia cells stably express in the cytoplasm a 46.5 kDa fusion protein, HMGB1: Lucia, in which the C-terminus of HMGB1 is fused to the Lucia luciferase. THP1-HMGB1-Lucia cells were used to monitor HMGB1 release. Briefly, THP1-HMGB1-Lucia cells were cultured in 200 μL X vivo media (∼200,000 cells) per well of a round-bottom clear culture 96-well plate (Corning 3799). Cells were treated in different concentrations of beQS-21 or ccQS-21 with or without 0.1 μg/mL LPS for 5 h. Supernatant (20 μL) were collected into flat-bottom white assay 96-well plates (Corning Costa 3922). After adding 50 μL of Quanti-Luc reagent (Invivogen, France), luminescence was detected using an EnVision Multimode plate reader (PerkinElmer, US).

#### IL-1β reporter assay

HEK-Blue IL-1β cells were used to monitor IL-1β secretion in THP1 cell lines with different gene editing upon inflammasome activation. Briefly, THP1 cell lines were cultured in 200 μL X vivo media (∼200,000 cells) per well of a flat-bottom clear culture 96-well plate (Corning 3599). Cells were first treated with 0.1 μg/ml LPS (Sigma, US) for 3.5 h and then the LPS media was replaced with fresh X vivo media. After LPS priming, cells were treated with different concentrations of beQS-21 or ccQS-21, for another 16 h. 20 μL of detected media with 180 μL HEK-Blue IL-1β cells (∼50,000 cells) per well were cultured in a flat-bottom clear culture 96-well plate (Corning 3599) for 20 h. Then, 20 μL of supernatant were collected into flat-bottom clear assay 96-well plates (Corning Costa 3361), After adding 180 μL Quanti-Blue reagent (Invivogen, France) and incubating at 37°C for 90 min, SEAP levels were detected using a SpectraMAX plate reader (Molecular Devices, US) at 655 nm.

#### Cytokine detection by Luminex

Cytokines in mouse air pouch fluid, blood, and cell culture media were detected by Luminex multiplex platform FLEXMAP 3D employing xPONENT software (Luminex, US). ProcartaPlex mouse and human cytokine panel kits from Invitrogen were used according to manufacturer’s instruction. Briefly, after adding and washing magnetic beads, samples and standards were added and incubated in dark with shaking either for 2 h at room temperature or overnight at 4°C. After wash, the detection antibody mixture was added for a 30-min incubation with shaking at room temperature, followed by washing and streptavidin-PE incubation with shaking at room temperature for 30 min. After incubation, beads were washed and reading buffer was added for cytokine detection.

#### ELISA assay for anti-OVA, gE and NANP IgG in mouse plasma

OVA/gE-specific antibody (IgG2c, IgG1, IgG) concentrations in plasma samples were quantified by ELISA using Chondrex ELISA kits. Briefly, 96-well plates were coated with antigen OVA/gE overnight at 4°C. Plates were washed and blocked for 1 h at room temperature. After washing, diluted samples and standards were then incubated for 2 h at room temperature. Plates were washed and incubated for 1 h at room temperature with HRP-conjugated anti-mouse IgG, IgG1, or IgG2c. Plates were washed and developed with TMB solution and then stopped with stop buffer. OD were read at 450 nm with an EnVision Multimode plate reader (PerkinElmer, US). Concentrations were calculated based on regression analysis of logOD and logConcentration plot. IgG2c/IgG1 ratio was also calculated. Anti-NANP Ig detection experiments were conducted at the Jenner Institute (Oxford, UK); Nunc Maxisorp plates were coated overnight at 4°C with 50μL of 2 mg/mL NANP_6_C peptide (Mimitopes) diluted in carbonate bicarbonate buffer. Plates were blocked for 1 h at room temperature with 1% BSA in PBS-Tween, samples were diluted in 1% BSA-PBS-Tween and incubated for 2 h at room temperature. Following washing, bound antibodies were detected by addition of alkaline phosphatase (AP)-conjugated goat anti-mouse IgG (Sigma-Aldrich) for 1 h at room temperature and addition of *p*-nitrophenyl phosphate, disodium salt substrate (Sigma-Aldrich). An arbitrary number of ELISA units (EU) were assigned to anti-NANP IgG monoclonal Ab (2A10) and optical density values of each dilution were fitted to a 4-parameter logistic curve using SOFTmax PRO software. ELISA units were calculated for each sample using the optical density values of the sample and the parameters of the standard curve.

#### Splenocyte restimulation assay for T_H_1/T_H_2 analysis

Immunized mice were euthanized 2 weeks after the boost or 4 weeks after prime immunization. Spleens were collected for splenocyte isolation. Splenocytes were passed through 70 μm cell strainers and suspended in RPMI supplemented with 2% FBS. Cells were collected after centrifuge and incubated with 3 mL of ACK lysis buffer (Gibco) for 5 min at room temperature to lyse red blood cells. Splenocytes were washed 2 times with RPMI supplemented with 2% FBS. Cells were then plated in flat-bottom culture 96-well plates (0.5 × 10^6^ cells per well). gE overlapping peptide mix (0.25 μg/mL, JPT Peptide Technologies, Germany), or OT1 and OT-II peptide mix (5 μg/mL, GenScript, China) were added for restimulation. After 48 h, supernatants were harvested for multiplexed T_H_1-2-17 cytokine analysis by Luminex using the Procarta-plex kits (Invitrogen, US).

#### IFNγ ELISpot

ELISpot assays were performed with mouse IFNγ ELISpotPLUS kits according to the manufacturer’s instructions (BD, US). A total of 0.5 × 10^6^ splenocytes were *ex vivo* restimulated with overlapping gE PepMix (0.25 μg/mL, JPT Peptide Technologies, Germany), or OT1 and OT-II peptides (5 μg/mL, GenScript, China), or E7 (5 μg/mL), or concanavalin A (5 μg/mL, Sigma, US). After overnight restimulation, plates were developed, washed and dried. Then spots were counted by an ELISpot plate reader (ImmunoSpot, US). Quality control was performed to remove background and make accurate counting of spots.

#### Cross presentation assays

Before the treatment day, Raw264.7-H2Kb cells were pretreated with recombinant murine GMCSF (400 U/mL = 20 ng/mL, Peprotech, US) overnight. GMCSF was also present in the treatment media. On the treatment day, cells (0.1 × 10^6^ per well in 96-well plates) were incubated with 1000 μg/mL ovalbumin (Ovalbumin EndoFit, Invivogen, France) or 10 μg/mL OT1 long peptides (Agenus, US) or 10 μg/mL E7 peptides (Agenus, US) or 10 μg/mL SIINFEKL (GenScript, China) and the indicated adjuvants as well as inhibitors for 6 h in a 37°C incubator. Then media was removed. Cells were washed and stained with an anti-mouse H-2Kb bound to SIINFEKL antibody (Clone 25-D1.16, Cat 141604, BioLegend, US) as well as LIVE/DEAD Fixable Near-IR Dead Cell Stain reagent (ThermoFisher, US) for flow cytometry analysis.

#### Jurkat-OT1-NFAT-luc reporter cell coculture assay

Raw264.7-H2Kb cells were primed and conditioned the same as above in culture-treated white 96-well plates, media was removed, and cells were washed for at least six times with PBS containing 0.1% BSA. The same number of Jurkat-OT1-NFAT-Luc reporter cells were added and co-cultured with Raw264.7-H2Kb for 20 h. Recombinant murine GMCSF (400 U/mL = 20 ng/mL) was added in the co-culture media. After incubation, the same volume of Nano-Glo luciferase substrate (Promega, US) was added and mixed with cell suspension for luminescence detection.

#### Mouse OT1 T cell proliferation assay

Raw264.7-H2Kb cells were primed and conditioned the same as above in culture-treated 96-well plates, media was removed, and cells were washed for four times with PBS containing 0.1% BSA. Cells were fixed with 1% glutaraldehyde, following washing with 1M Glycine 2X and PBS containing 0.1% BSA 2X. The same number of CellTrace (Invitrogen, US)-stained mouse OT1 T cells were added and co-cultured for 3 days. Recombinant murine IL-2 (50 U/ml) was added in the co-culture immune cell media. Cells were washed and stained with an anti-mouse CD11b (BUV805, Clone M1/70, BD), anti-mouse CD3e (BUV395, Clone 145-2C11, BD), anti-mouse CD8 (BUV563, Clone 53–6.7, BD), anti-mouse CD4 (NobaBlue610, Clone GK1.5, Invitrogen), as well as LIVE/DEAD Fixable Near-IR Dead Cell Stain reagent (ThermoFisher, US) for flow cytometry analysis.

#### Pseudo cross presentation assays

Raw264.7-H2Kb cells (0.1 X 10^6^ per well in 96-well plates) were treated with or without 50 ng/mL Bafilomycin A1 (SelleckChem, US) as inhibitor, and then treated with toxin (either 25 ng/mL Saporin (Sigma, US) or 2.5 mg/mL Cytochrome *c* (Sigma, US)) in the presence of the indicated adjuvants for 5 h at 37°C. Then, media was removed, and cells were washed with PBS. Fresh media was added, and cells were incubated overnight. Supernatant LDH as cell death indication was analyzed by CytoTox 96 Non-Radioactive Cytotoxicity Assay (Promega, US).

### Quantification and statistical analysis

A two-tailed Student’s *t* test or a one-way analysis of variance (ANOVA) was performed when comparing two groups or more than two groups, respectively. Statistical analysis was performed using Microsoft Excel and Prism 9.0 (GraphPad). Data are expressed as means ± s.d. Difference was considered to be significant if p < 0.05 (ns: non-significant, ∗: p < 0.05, ∗∗: p < 0.01, ∗∗∗: p < 0.001, ∗∗∗∗: p < 0.0001 unless otherwise indicated).
