# Supplementary material for: Chemical and biological characterization of vaccine adjuvant QS-21 produced via plant cell culture
Source: iScience. 2024 Jan 26;27(3):109006. doi: 10.1016/j.isci.2024.109006 (PMC10867646; doi:10.1016/j.isci.2024.109006)
Supplement: Document S1. Figures S1–S6 and Tables S1–S3 [file mmc1.pdf]

iScience, Volume *27*

Supplemental information

Chemical and biological characterization of vaccine adjuvant QS-21 produced via plant cell culture

Xiangmin Lv, Jesse Martin, Heather Hoover, Bishnu Joshi, Margaret Wilkens, David A. Ullisch, Thomas Leibold, John S. Juchum, Sanket Revadkar, Barbara Kalinovska, Justin Keith, Adam Truby, Gui Liu, Elaine Sun, John Haserick, Jon DeGnore, Joseph Conolly, Adrian V.S. Hill, John Baldoni, Charlotte Kensil, Dan Levey, Alexandra J. Spencer, Gilbert Gorr, Mark Findeis, and Antoine Tanne

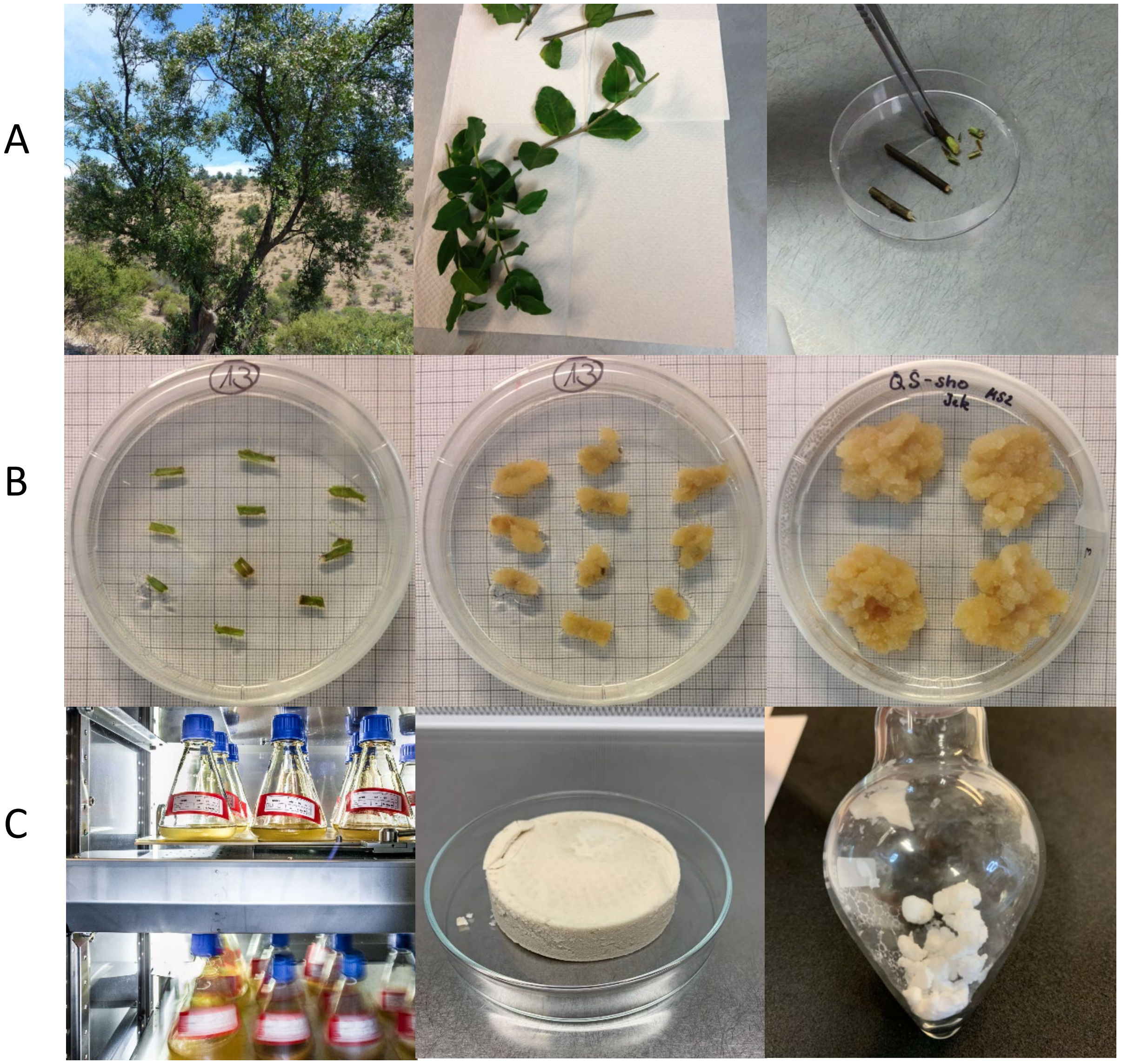

Figure S1: Representation of key initial steps which led to demonstration of the feasibility to produce QS-21 from cell culture related to Figure 1. (**A**) Authenticated wild-grown cuttings of mature healthy *Quillaja saponaria* (QS) trees were harvested in the Andean foothills in the vicinity of Santiago, Chile. (**B**) Explants from stems were grown on solid medium to generate callus cultures. (**C**) Calli were transferred to liquid medium to produce suspended mixed cell cultures. Suspension cultures were grown and induced to obtain biomass which was extracted. The application of a modified downstream serial RP-HPLC process based on the one developed by Agenus Inc. for producing beQS-21 for clinical studies was used to complete the isolation of high purity plant cell culture derived QS-21 from a complex mixture of saponins present in the biomass. This material was designated as ccQS-21 in this study.

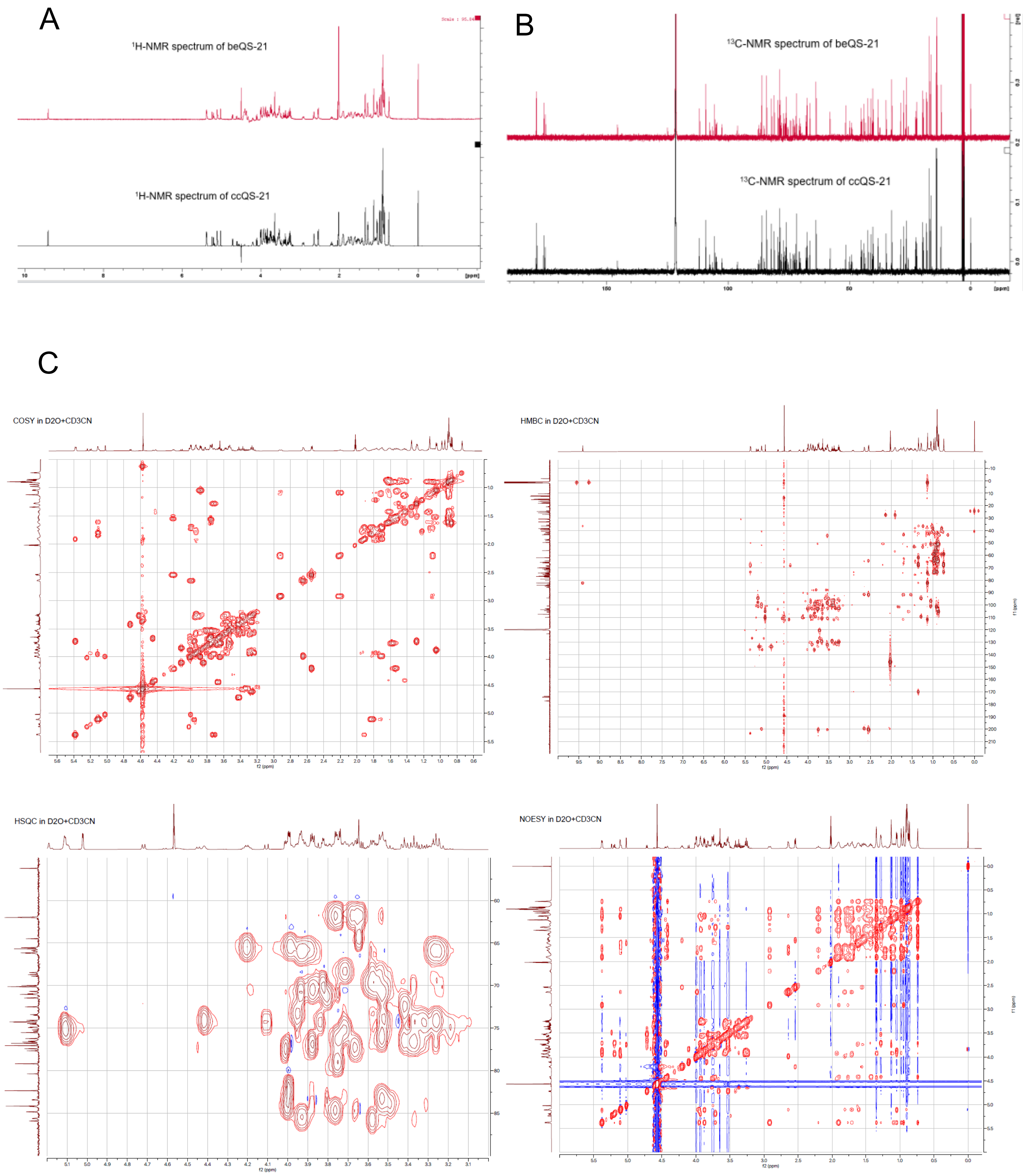

Figure S2. Additional analytical comparison of ccQS-21 and beQS-21 demonstrating similarity in complement to Figure 2. (**A**) ^{1}H-NMR spectra of cell culture QS-21 (ccQS-21) and bark extract (beQS-21). (**B**) ^{13}C-NMR spectra of ccQS-21 and beQS-21. Both ^{1}H and ^{13}C-spectra of ccQS-21 show the same structural features as beQS-21. The head-to-head comparison of ^{1}H-NMR spectrum at 600 MHz and ^{13}C-NMR spectrum at 150 MHz confirm that both cc and be QS-21 are structurally comparable. **(C)** 2D-NMR spectra of ccQS-21, COSY, HMBC, HSQC, and NOESY further demonstrate the structure of plant cell culture QS-21 and confirm its structural identity.

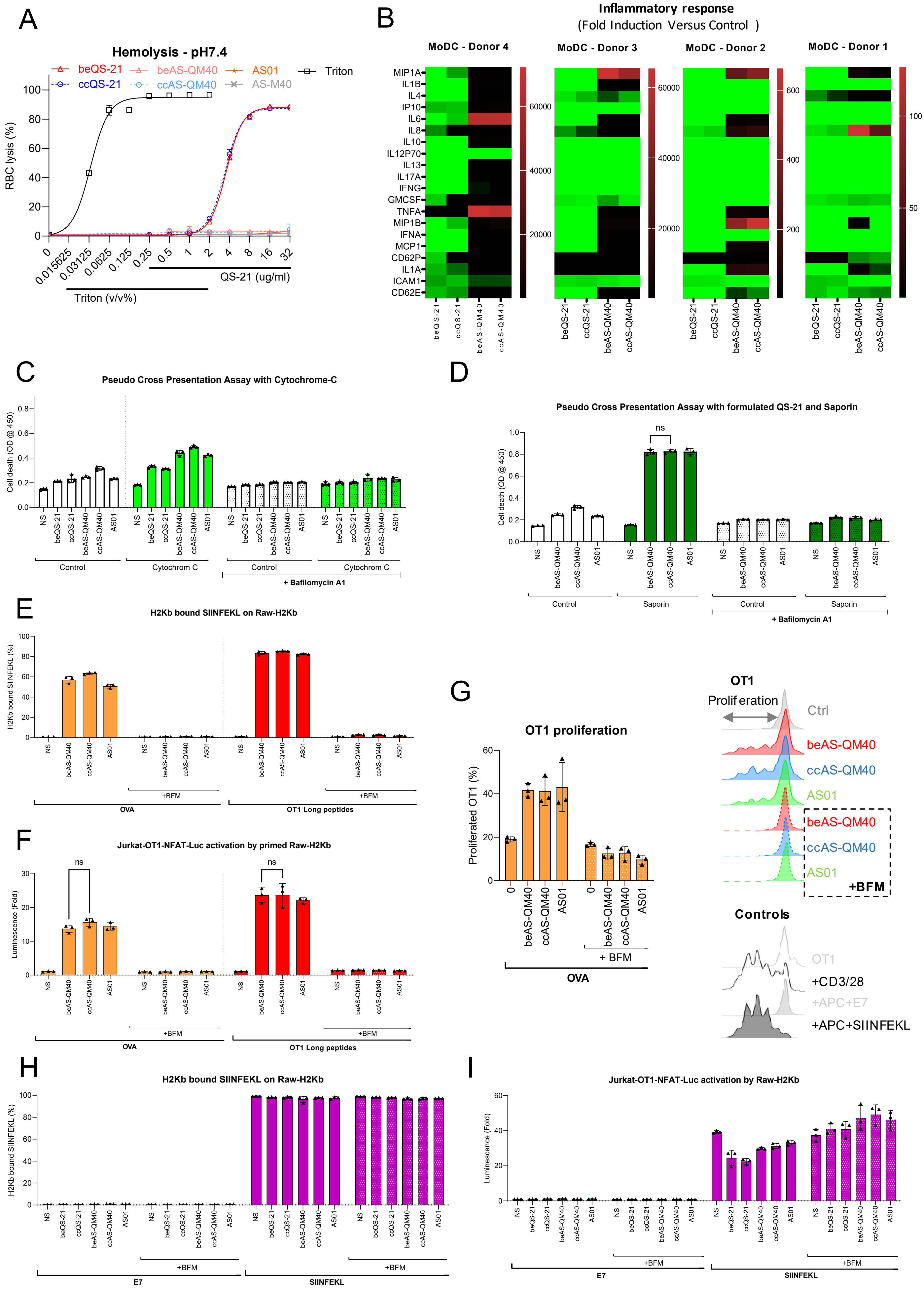
A
Hemolysis - pH7.4
beQS-21
ccQS-21
beAS-QM40
ccAS-QM40
AS01
AS-M40
Triton
RBC lysis (%)
QS-21 (ug/ml)
Triton (v/v%)
B
Inflammatory response
(Fold Induction Versus Control)
MoDC - Donor 4
MoDC - Donor 3
MoDC - Donor 2
MoDC - Donor 1
MIP1A
IL1B
IL4
IP10
IL6
IL8
IL10
IL12P70
IL13
IL17A
IFNG
GMCSF
TNFA
MIP1B
IFNA
MCP1
CD62P
IL1A
ICAM1
CD62E
C
Pseudo Cross Presentation Assay with Cytochrome-C
Cell death (OD @ 450)
Control
Cytochrom C
+ Bafilomycin A1
D
Pseudo Cross Presentation Assay with formulated QS-21 and Saporin
Saporin
E
H2Kb bound SIINFEKL on Raw-H2Kb
H2Kb bound SIINFEKL (%)
+BFM
OVA
OT1 Long peptides
F
Jurkat-OT1-NFAT-Luc activation by primed Raw-H2Kb
Luminescence (Fold)
G
OT1 proliferation
Proliferated OT1 (%)
+ BFM
OVA
OT1
Proliferation
Ctrl
Controls
OT1
+CD3/28
+APC+E7
+APC+SIINFEKL
H
H2Kb bound SIINFEKL on Raw-H2Kb
E7
SIINFEKL
I
Jurkat-OT1-NFAT-Luc activation by Raw-H2Kb

Figure S3. Additional characterization related to Figure 3; demonstrating that both QS-21 retain the equivalent biochemical and biological properties when formulated in AS01 mimetic liposomes. (**A**) The formulation of both beQS-21 and ccQS-21 with liposomes containing the same ratio of molecules of cholesterol to QS-21 abrogate the hemolytic properties of QS-21 to a similar undetectable level identical to AS01. (**B**) Formulated ccQS-21 and beQS-21 can induce the same innate inflammatory response. Monocyte-derived Dendritic Cells (MoDCs) derived from four different donors display the same profile of inflammatory response when they are stimulated with ccQS-21 and beQS-21 formulations as measured by a multiplexed cytokine quantification method. (**C-D**) Both ccQS-21 and beQS-21 formulated in liposomes can enhance the leakage of antigens from the endo phagolysosomal compartment to the cytoplasm which highly resemble the initial key step associated with antigen cross-presentation in antigen-presenting cells (APC). Using a viability-based pseudo-cross presentation assay, formulated ccQS-21 and beQS-21 were shown to equivalently induce the cytosolic translocation of indicated toxins leading to an increase cell death as compared to QS-21 alone or toxin alone. (**E)** In another cross-presentation assay flow cytometry analysis was used to assess the MHC-I epitope cross-presentation using a TCR-like antibodies (H2kb-SIINFEKL). Both ccQS-21 and beQS-21 formulations can comparably induce the antigen translocation to cytosol and cytoplasmic processing of antigens to enhance their presentation on MHC-I at the surface of APCs. (**F)** The ability of the primed APCs to activate T cell response was then further assessed using a transgenic reporter NFAT luciferase T cell line expressing the relevant murine transgenic OT1 TCR. Both beQS-21 and ccQS-21 formulations equivalently enhance the APCs ability to activate the reporter system and trigger the TCR activation in MHC-I peptide specific manner. (**G)** Both ccQS-21 and beQS-21 formulations equivalently enhance the cross-presentation by APCs to induce primary OT1 T cell activation and proliferation. **(C-G)** The sensitivity to the bafilomycin A (BFM) demonstrate that antigen cross presentation induced by both QS-21 is dependent on phagolysosome maturation. **(H-I)** When stimulated with minimal MHC-I epitope SIINFEKL; saponins cannot enhance antigen presentation and this is not regulated by the suppression of lysosome acidification.

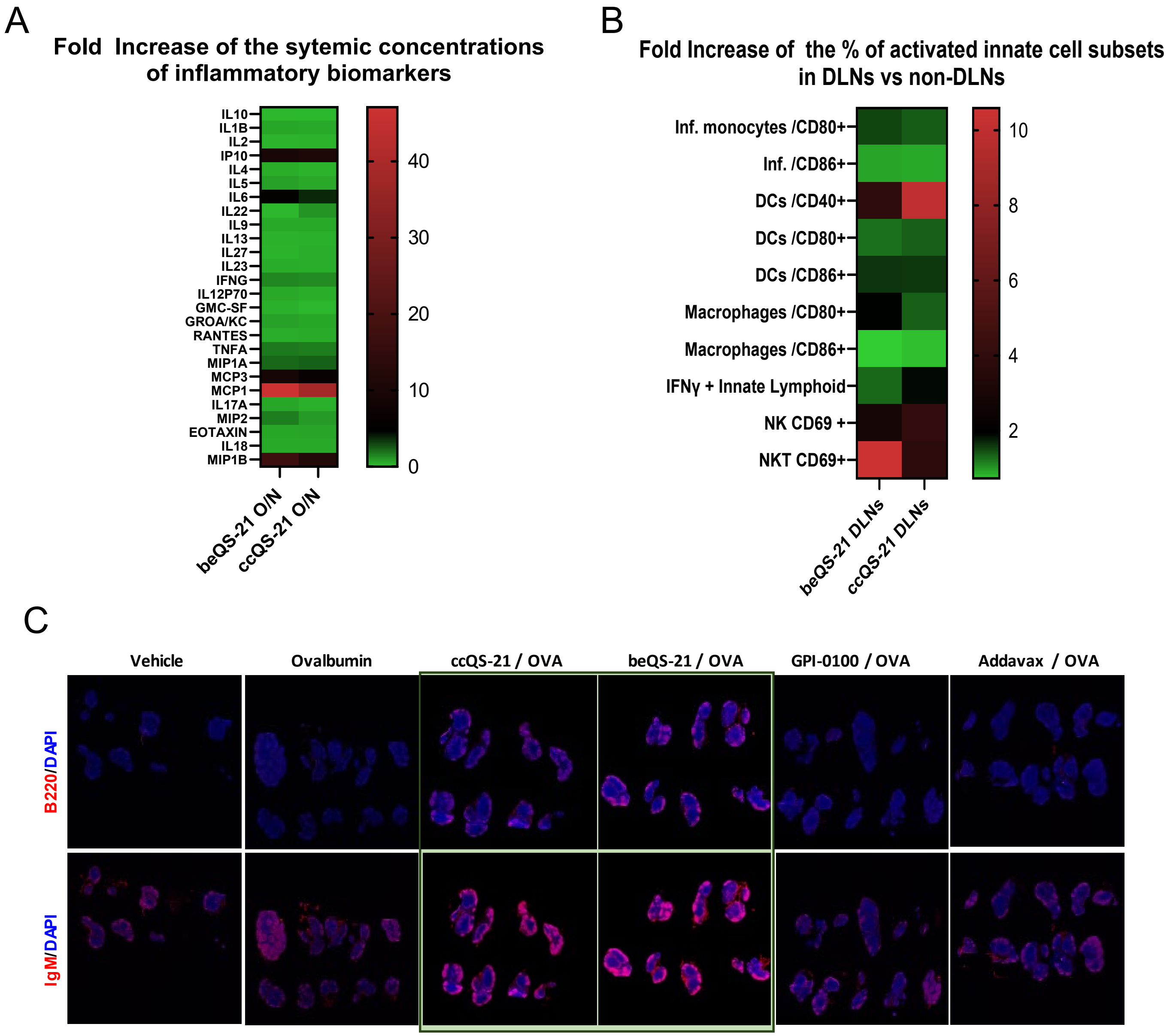

Figure S4. Additional Immune Phenotyping in complement to Figure 4, demonstrating both QS-21 induce the same innate immune response priming. (**A**) Both ccQS-21 and beQS-21 induce the same profile of systemic inflammation mostly associated with the recruitment of leukocyte and particularly myeloid cells. (**B**) The extensive immune phenotyping by flow cytometry of draining lymph nodes (DLNs) and non- draining lymph nodes (NDLNs) after overnight exposure to the adjuvant confirmed that both beQS-21 and ccQS-21 can induce a broad innate immune myeloid and lymphoid cell recruitment and activation in the vaccinal DLNs. **(C)** Immune phenotyping by multiplex fluorescent Immunohistochemistry (IHC) of DLNs after overnight exposure to adjuvants revealed that both beQS-21 and ccQS-21 comparably enhance the activation of B cells in the germinal centers.

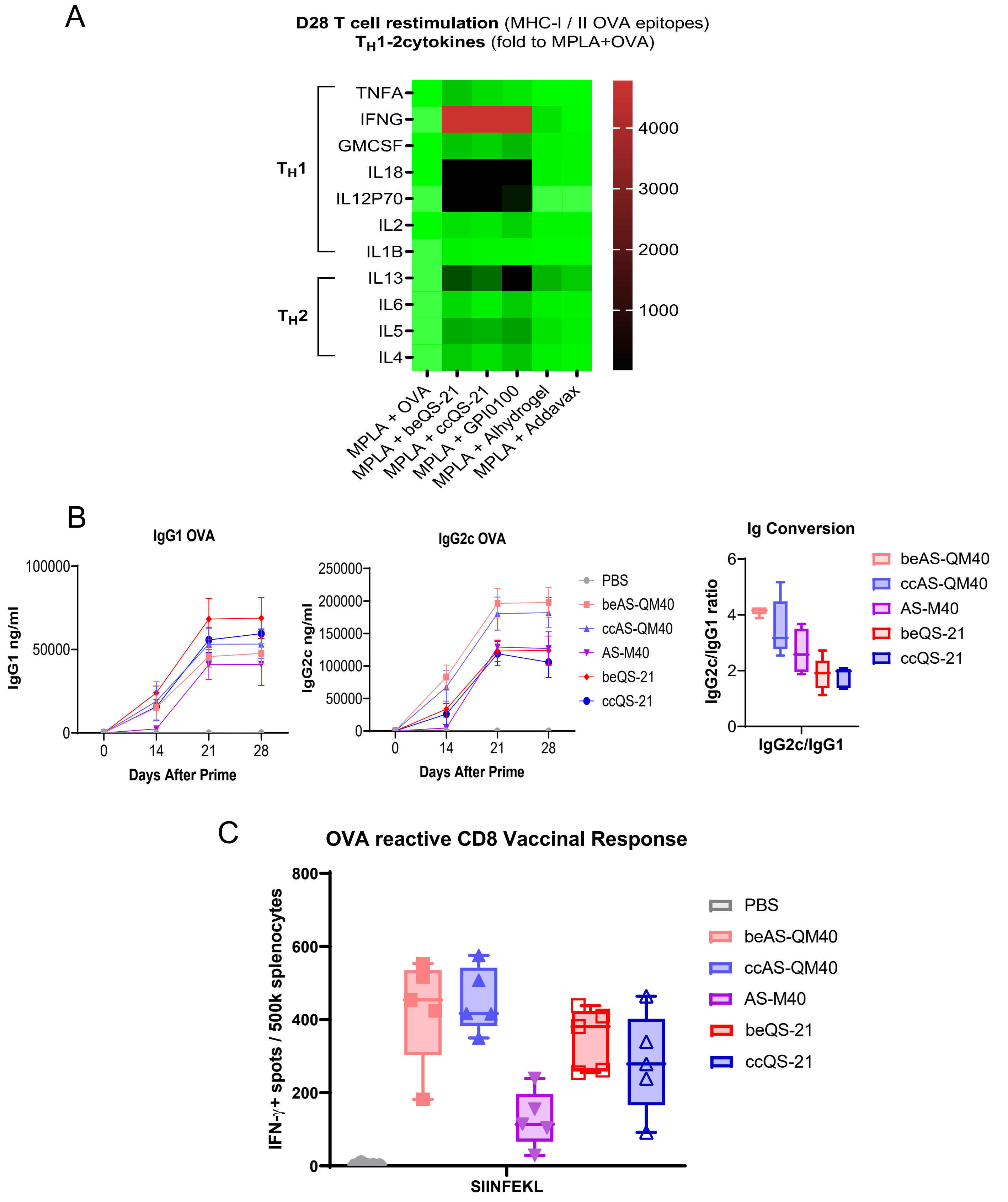

Figure S5: Additional characterization related to Figure 5 demonstrating that the TLR4 agonist, MPLA can enhance the adjuvant properties of both QS-21. (A) Multiplexed analysis of the profile of the T cell vaccinal response upon *ex vivo* restimulation with a mixture of MHC-I/II ovalbumin epitopes and measured by the fold increase production of $T_{H}1$ and $T_{H}2$ cytokines as compared to the reference vaccines adjuvanted with MPLA alone. Both ccQS-21 and beQS-21 synergize with MPLA such as the other saponin-derived adjuvant GPI-0100 and induces a $T_{H}1/2$ cytokine profile as compared to references adjuvant. This was further demonstrated using liposome co-formulations of QS-21 and MPLA **(B-C)**. Both ccQS-21 and beQS-21 enhance the adjuvant potency of MPLA-containing liposomes and increases the $T_{H}1$-biased humoral and T cell activation properties.

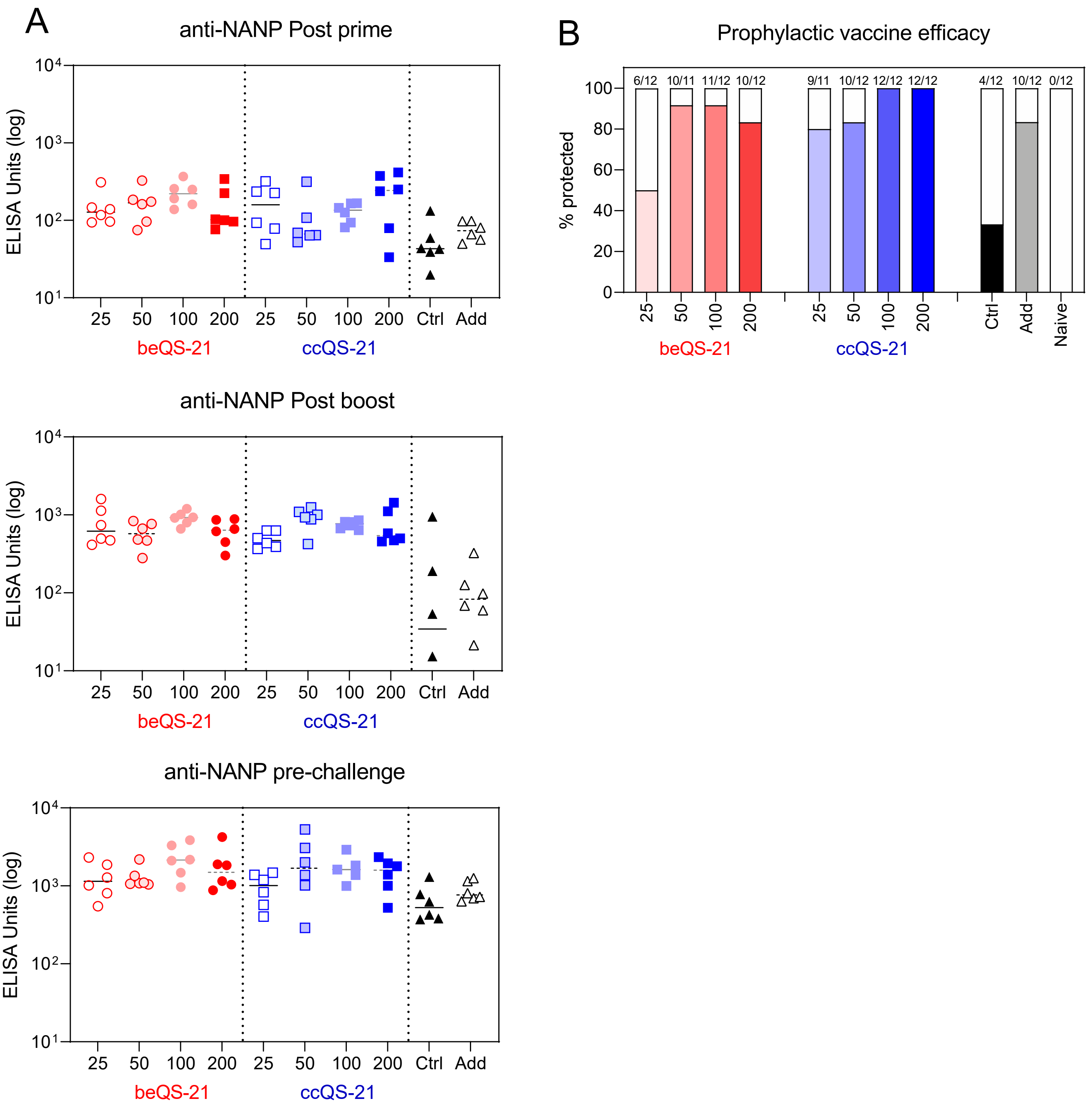

Figure S6: Complementary data related to Figure 6 demonstrating that both QS-21 enhance MPLA adjuvant properties. Using the R21 anti-malaria vaccine efficacy model in mice, the equivalence of ccQS-21 and beQS21 was further demonstrated through an adjuvant dose sparing approach in which the dose of QS-21 can be reduced without significantly affecting the adjuvant properties. Both ccQS-21 and beQS-21 retain maximal adjuvant potency based on (**A**) immunogenicity and (**B**) efficacy monitoring from a range of 2.5 µg to 10 µg of QS-21 co-formulated with 2 µg of MPLA injected per dose per animal. The lower dose of 1.25 µg of QS-21 may induce slightly more protective trends but remains highly potent regarding the immune response priming. All tested doses remain superior to the MPLA-control liposome.

Table S1: Gradient for HPLC analysis related to the STAR Methods.

Time (minutes)	%A	%B	Flow Rate (mL/min)
0.0	70.0	30.0	1.00
30.0	55.0	45.0	1.00
31.0	10.0	90.0	1.00
32.0	70.0	30.0	1.00
32.5	70.0	30.0	1.50
47.5	70.0	30.0	1.50
48.0	70.0	30.0	1.00

Table S2: TripleTOF mass spectrometry settings related to the STAR Methods.

TOF MS Parameters	
Polarity:	Positive
Accumulation Time:	250.0 ms
Time Bins to Sum	4
Curtain Gas	30
Ion source Gas1	45
Ion source Gas2	50
Ion Spray Voltage Floating	5500
Temperature (˚C)	450
Collision Energy	10
Declustering Potential	180
MS TOF m/z range	400-2500
TOF MS/MS	
Polarity:	Positive
Accumulation Time:	100.0 ms
Curtain Gas	30
Collision Energy	10
Collision Energy Spread	20
Declustering Potential	180
Product Ion TOF m/z range	100-2000
Exclude for	10 sec
Exclude after occurences:	3

Table S3: QS-21 liposome formulation description related to the STAR Methods.

Formulation description	QS-21	QS-21 µg/mL	MPLA type	MPLA µg/ml
AS01	Bark extract from GSK	100	3D MPL	100
AS-QM40 (AS-Q100M40)	Bark extract and cell culture from Agenus and SaponiQx	100	PHAD	40
AS-Q25M40	Bark extract and cell culture from Agenus and SaponiQx	25	PHAD	40
AS-Q50M40	Bark extract and cell culture from Agenus and SaponiQx	50	PHAD	40
AS-Q200M40	Bark extract and cell culture from Agenus and SaponiQx	200	PHAD	40
AS-M40	N/A	0	PHAD	40
